# A generic schema and data collection forms applicable to diverse entomological studies of mosquitoes

**DOI:** 10.1186/s13029-016-0050-1

**Published:** 2016-03-28

**Authors:** Samson S. Kiware, Tanya L. Russell, Zacharia J. Mtema, Alpha D. Malishee, Prosper Chaki, Dickson Lwetoijera, Javan Chanda, Dingani Chinula, Silas Majambere, John E. Gimnig, Thomas A. Smith, Gerry F. Killeen

**Affiliations:** Environmental Health and Ecological Sciences Thematic Group, Ifakara Health Institute, P.O. Box 53, Ifakara, Tanzania; Department of Mathematics, Statistics and Computer Science, Marquette University, Milwaukee, WI 53201-1881 USA; Pacific Malaria Initiative Support Centre, School of Population Health, University of Queensland, Brisbane, 4006 Australia; Vector Biology Department, Liverpool School of Tropical Medicine, Pembroke Place, Liverpool, L3 5QA UK; National Malaria Control Centre, Lusaka, Zambia; Division of Parasitic Diseases, Centers for Disease Control and Prevention, Atlanta, GA USA; Department of Public Health and Epidemiology, Swiss Tropical Institute, Socinstrasse 57, Basel, CH 4002 Switzerland

## Abstract

**Background:**

Standardized schemas, databases, and public data repositories are needed for the studies of malaria vectors that encompass a remarkably diverse array of designs and rapidly generate large data volumes, often in resource-limited tropical settings lacking specialized software or informatics support.

**Results:**

Data from the majority of mosquito studies conformed to a generic schema, with data collection forms recording the experimental design, sorting of collections, details of sample pooling or subdivision, and additional observations. Generically applicable forms with standardized attribute definitions enabled rigorous, consistent data and sample management with generic software and minimal expertise. Forms use now includes 20 experiments, 8 projects, and 15 users at 3 research and control institutes in 3 African countries, resulting in 11 peer-reviewed publications.

**Conclusion:**

We have designed generic data schema that can be used to develop paper or electronic based data collection forms depending on the availability of resources. We have developed paper-based data collection forms that can be used to collect data from majority of entomological studies across multiple study areas using standardized data formats. Data recorded on these forms with standardized formats can be entered and linked with any relational database software. These informatics tools are recommended because they ensure that medical entomologists save time, improve data quality, and data collected and shared across multiple studies is in standardized formats hence increasing research outputs.

**Electronic supplementary material:**

The online version of this article (doi:10.1186/s13029-016-0050-1) contains supplementary material, which is available to authorized users.

## Background

To understand the dynamics of vector-borne diseases such as malaria, empirical data is required to develop an in-depth knowledge of relevant ecology, genetics, risk factors, infection rates, and clinical outcomes [1, 2]. The leading vector control strategies (i.e., long-lasting insecticidal nets (LLINs) and indoor residual spraying (IRS)) can reduce indoor malaria transmission, but these tools alone are insufficient to eliminate malaria, especially from intensely endemic regions [3–5]. To achieve malaria elimination, control of indoor transmission with LLINs and IRS must be improved [6–10] and supplemented with vector control strategies that target adult mosquitoes outdoors or at source in their aquatic habitats [3–5, 11]. To develop and evaluate interventions for malaria, especially new ones designed to exploit the ecology of target species, a holistic and multidisciplinary approach is necessary, with multiple researchers collaborating, sharing, and synthesising data across multiple studies and laboratories.

Standardised data schema, ontologies and databases have been used across many scientific fields from genetics to epidemiology and, more recently, ecology to improve scientific output. If well-structured, user-friendly, consistently applicable informatics tools are adopted early in the research process, individual researchers and the broader research community accumulate increased benefits over the long term, including reduced time from data collection to dissemination, facilitation of data sharing, streamlining of multisite collaborations, and enhanced retrospective analysis [12–17]. Standardised data schema, databases and even public data repositories exist for genetic data for malaria parasites and for their human and mosquito hosts [18, 19], and similar controlled and standardised systems are available for epidemiological studies of malaria-infected human beings. However, equivalent systems for studies of the live mosquitoes which mediate transmission are only now emerging [20, 21].

Significant challenges are presented by the variety of data formats, ecological structures, experimental designs and sampling methods used in studies of mosquitoes, which often collect very large volumes of data and adaptively change experimental design over periods as brief as months, weeks or even days. Despite this level of data complexity and variability, experimental and survey data describing mosquitoes often are collected using experiment-specific forms that require frequent, error-prone redesign. Such cursory data management leads to badly or inconsistently structured data, frequent transcription errors, difficulty in sharing or linking data and information loss [22]. Improved informatics tools for malaria vector studies are required to provide structure to data at the point of data collection and streamline use of databases that consistently link field and laboratory data. Therefore, we have developed a generic schema for recording taxonomic, abundance and phenotypic data, as well as processing associated samples, derived from surveys of malaria vectors caught in the field or manipulated in enclosed experimental systems. These tools were developed specifically for application in lower-income tropical countries with limited access to specialized software and expert informatics support.

## Methods

**Fig. 1:**
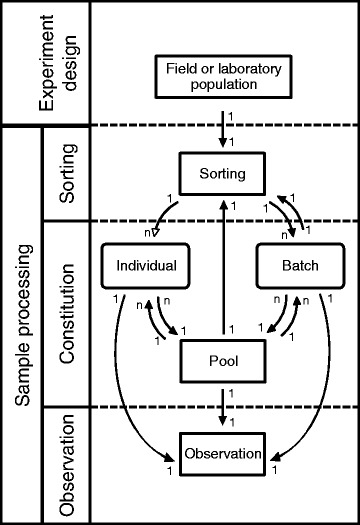
Data collection is based on a simple foundation of recording the experimental design followed by sample processing. Sample processing (dashed boxes) involves the sorting and observation of mosquito samples. As mosquito biology experiments are highly variable in structure, there are many possible ways in which to move between the generic schemas. The arrows indicate the direction and function (e.g., one-to-many: 1…n) of the relationships between the entities

In keeping with the goal of making this system widely available and practicable in resource-limited developing countries, all forms and data dictionary are available as Microsoft Excel® templates **[**see Additional file 1], which were used in accordance with the standard operating procedures document [see Additional file 2]. Some users subsequently entered the recorded data using specially tailored applications on laptops or mobile devices chosen and implemented at their own discretion. However, most data entered directly into tables in Excel® structured consistently with the generic schema described in Fig. 1, using the attribute names from the forms as headers so that they could be imported into readily available relational database software, for example Micrsoft Access® or My SQL, and then linked and cleaned using the primary and alternative keys described below.

### A generic schema for recording data from mosquito surveys and experiments

Although research in mosquito biology involves very large number of possible experimental and survey procedures, the vast majority can be described within a single fundamental structure (Fig. 1). Essentially, each experiment commences with a defined *experimental design*, followed by *sample collection, sorting, constitution,* and *observation*.

#### Sample collection

A *collection* is defined as a group of mosquitoes from one sampling or trapping effort. The mosquitoes could be at any stage in the life cycle (i.e., egg, larvae, pupae, or adult) and are collected from a natural population in the field or from a captive insectary/semi-field colony. It is critically important to know where, when, and how each collection was executed [23, 24], so these experimental or survey design attributes must be recorded before or immediately after each is completed. In some cases, mosquitoes are collected in the context of an *experiment* in the true sense, meaning that the field or laboratory environment the mosquito population lives in is deliberately manipulated in some way to measure either the effect of the manipulation or to reveal specific phenotypic responses to those manipulations. However, in other cases, collections within the context of a *survey* merely obtain samples of the mosquito population without any deliberate manipulation of that population by the researcher.

#### Sample sorting

After the collection of mosquitoes is made, the researcher sorts it on the basis of specific, directly observed attributes. A sample *sort* is defined as the process by which a collection or sample is broken into subgroups on the basis of specific categorical attributes defined by direct observation at the point of collection, with or without specific experimental manipulations to reveal specific phenotypes. For example, a collection of mosquitoes from one trapping effort can be broken into subgroups of pre-defined taxon, sex, and abdominal status, and the number in each subgroup is observed by counting. In fact, most experiments that are conducted by entomologists generally, and mosquito biologists in particular, rely on sorting samples into pre-defined categories based on the observed attributes of individual insects. While this sorting process is almost always followed by counting of mosquitoes in each category, this enumeration is a subsequent observation of the sample that is distinct from those observations used to define and prepare it by sorting. The observed attributes used to sort collections of wild-caught insects always include some level of taxonomic classification. For mosquitoes, it is also typical to include their sex and abdominal status. Experimental manipulation of captive or wild mosquitoes also may be used to enable sorting based on classification of specific response phenotypes. A common example is a 24-h survival analysis of mosquitoes after they have been exposed to an insecticide [25]: the researcher sorts the mosquitoes by ‘dead’ or ‘alive’ after completion of the 24-h holding period, and the number of mosquitoes in each subgroup is then observed by counting.

The observations used to designate the sorting of mosquitoes into sub-groups must be recorded as attributes with continuous measurements classifying into categorical strata defined before conducting the experiment. Categorical sorting observations, such as alive versus dead within a sequence of pre-defined holding periods so their range of possible attributes values can be pre-filled into the sort form. Values for a continuous variable that is recorded based on scalar observations or measurements, such as time of copulation, may be directly observed and recorded as a continuous attribute during an experiment or analytical assay. However, such a continuous attribute cannot be used only to sort mosquitoes into samples containing single individuals unless pre-defined ranges of these measures are assigned as nominal or ordinal categories into which several insects can be classified. Alternatively, such continuous attributes may be recorded in ordinal, discontinuous format by either observing intermittently or measuring by assignment to specific strata with defined boundaries. For example, time of death is clearly a continuous quantity, but it may be recorded by removing dead insects over a sequence of exposure durations that need to be designated by the researcher before commencing the experiment.

#### Sample constitution

After the collection is sorted and the number of mosquitoes in each subgroup has been observed, the mosquitoes can be used to constitute samples as individuals or batches. An *individual* is defined as one mosquito, and a *batch* is a group of two or more mosquitoes created from one source collection. Individuals or batches may be merged together to form *pools* of mosquitoes that are defined as a group of mosquitoes assembled from more than one source individual, batch and/or collection.

#### Sample observation

An *observation* is a direct scientific observation of a defined attribute for a single whole sample, for example, the counted number of individuals in it. For individual mosquitoes, observation may include sibling species identification [26], blood-meal identification [27], sporozoite stage [28], ovarian dissection to determine gonotrophic age class [29, 30], or visual measurement of wing length [31]. Additionally, researchers may make observations of mosquito genotype [32] and then could link to the semantics of gene ontology [18] using complementary databases such as VectorBase [33].

A common mistake is to confuse the observed attributes used to define and prepare a sample by sorting with those assigned to that sample based on subsequent observations of it. This can be a difficult concept to grasp at first, and one that we commonly confused while designing this schema. However, the foundation of a *sort* is the process by which one sample (collection, batch or pool) is broken into many based on observation of categorical or continuous *sort attributes*, whereas a *sample observation* is a direct observation or measurement of a property of a single sample. For example, a knock-down insecticide assay of a batch or pool of mosquitoes begins with a sorting process, where the original sample is sorted into subgroups and samples based on observed survival attributes following a sequence of pre-defined holding periods. Afterwards, the number of individuals in each sorted subgroup is observed by counting them. However, this quantity is an attribute of that sample that is observed after it is prepared, rather than an attribute used to prepare it by sorting.

### Generic standardized data collection forms

The majority of entomological studies of tropical vector-borne diseases are conducted in lower-income countries, where access to specialized software and expert informatics support is often limited, so we designed a limited number of generic, standardized paper-based data collection forms. Our six categories of data collection forms are informed consent record (IC), experimental design (ED), sample sorting (SS), sample observation (SO), and sample storage (ST) [see Additional file 1 for the actual paper-based data collection forms]. The IC and ST forms are not novel and can be applied generically to recording details of informed consent for human participants and for sample storage location, in any type of study rather than just entomological ones. However, the ED, SS, and SO forms are designed specifically for recording the relevant details of entomological sample collection, sorting, observation, and constitution, respectively (Table 1). Within each category, there are up to three different form designs to accommodate a wide variety of experimental procedures, only one of which is required for a specific individual experiment. Each experiment commences with an experimental design (ED), followed by sample sorting (SS). The ED form can be just as readily applied to recording where, when and how mosquitoes are collected [23] as part of a survey of an un-manipulated population as it can to an experiment in which a population is deliberately manipulated. If required, additional forms can record further sample observations (SO), sample and storage (ST), as well as informed consent numbers for human participants (IC).

**Table 1:** Description of generic schema categories

Schema category	Table	Description	Unique identifier
Informed consent record	Informed consent record	Details of written informed consent forms	IC1
Experimental design	Field collections	Records the design of experiments collecting mosquitoes in the field	ED1
	Batch and/or pool experimental assay	Records the design of experiments using colony mosquitoes or pre-existing batches or pools	ED2
Sample sorting	Adult field collection	Records the process where a field collection of mosquitoes is sorted into pre-defined subgroups based on taxon, sex and abdominal status	SS1
	Immature field collection	Records the process where a field collection of mosquitoes is sorted into each specified combination of taxon and body-part (which incorporates developmental stage)	SS2
	Batch and/or pool experimental assay	Records the process wherein a batch and/or pool or mosquitoes is experimentally sorted into pre-defined categories	SS3
Sample observation	Laboratory analysis	Scientific observations made using laboratory analyses of mosquito samples	SO1
	Dissection and wing length	Scientific observations made to measure the parity status (females only) and wing length of dead individual mosquitoes	SO2
	Individual experimental assay	Various scientific observations of individual mosquitoes made in the field or entomology laboratory	SO3
	Box record	Records the long-term storage of sample boxes in the laboratory storage facility	ST

Details of the primary tables of the generic schema that are used as the foundation of the relational database and are reflected in the data collection forms

**Fig. 2:**
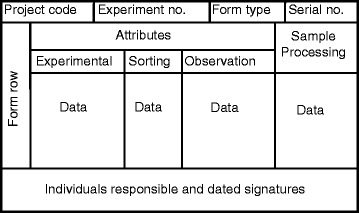
The generalised structure that was used as the foundation for designing each of the data collection form. This figure presents a generic structure used to design each of the data collection form. The top rows record information that uniquely identifies each form, central grid records the actual data and observations under each attributes. To preserve the integrity of the data, managing responsible personnel, and facilitating external audits both the supervisor and the responsible personnel can initial and sign the bottom section of the form

Each data collection form was designed using the same generic structure shown in Fig. 2. The top rows record the *project code, experiment number, form type and serial number* attributes that uniquely identify each form, as well as additional variables that are specific to each form type, such as ethical approval number (IC), study site (ED) or body part (SS). The actual data and observations are recorded in the central grid on the form. Listed along the top of the grid are the names of the various attributes that can be used to record the experimental design, sort criteria or direct observation. A comprehensive list of attributes has been created, a minority which are earmarked as mandatory for rigorous data collection. However, to provide flexibility to the user, most attributes are optional. Some attributes are termed generic because they are widely understood and accepted, so they can be used across all experiments in the same manner. However, experiment-specific also attributes are provided which are user-defined and only have context within the bounds of the experiment in question. The short, two or three-letter, capitalized acronym for each attribute should not be changed, it is used to label each attribute in the form and each variable in the electronic data table. However, the full names of each attribute can be edited in the form template for context-specific use, including translation into the local language, so long as the meaning of the edited version is not altered.

The response category for each attribute uses numerical codes because entering data in string format is usually slower and more error-prone. The generic attributes have standardized codes that are used by all users and are printed on the bottom or back of the forms, so it is preferable to record as many attributes as possible using these carefully standardized options to ensure comparability of data from different studies, teams or countries. Nevertheless, columns for experiment-specific attributes, which are not captured by the generic options, also allow the user to define codes for these additional variables. While some experiment-specific attributes, such as *experimental round, replicate* or *treatment*, are common features of diverse studies and are pre-filled as options available to the end-user, these can be over-written, and additional blank columns are also available for new user-defined attributes.

Auditable data and sample handling is very important, but often overlooked, in entomological research because many studies rely on the high fidelity exchange of samples and data between by distinct individuals, teams, and facilities responsible for distinct components of the process, working separately with correspondingly separate forms. Creating an auditable trail in the data record allows the user to move succinctly within the system and trace each datum and responsible individual back to the original document. Such an auditable data trail is essential for data cleaning, preserving the integrity of the data, managing responsible personnel, and facilitating external audits. The same principle is followed by financial accountants who need to be able to follow the trail from the balance sheet to individual voucher.

This is achieved by 1) the researcher clearly pre-entering the experimental design and specifying required attributes, and 2) at each stage in the experimental process, both the supervisor and the responsible personnel can initial and sign the bottom section (Fig. 2). The bottom section of the forms records transfer of sample handling and decision-making responsibilities between individuals at each point in the experiment, thus creating a clear chain of communication and accountability for all responsible personnel. An auditable trail for the data and samples themselves is created with a unique identifier, termed *serial number,* at the top of each form and unique *row* numbers to identify the individual components of the data. Thus within an experiment, each row of data can be identified uniquely using the minimum amount of information, specifically the combination of the *form serial* and *row* numbers. Many ED forms are completed in each experiment, each line of which results in completing an associated SS form, and optionally, additional SO and ST forms also may be associated with the SS form. For any pair of associated forms, the *source form* is defined as the form which defines the composition of a collection or sample, while the *destination form* is defined as a subsequent form describing the next sort, observation or re-constitution step. As an example, for any associated pair of ED and SS forms, the ED is the source form for the SS form data, while SS is the destination form for the ED form data but represents the source form for any SO, or ST destination forms recording subsequent sample observation, constitution or storage data. To provide an identifier that uniquely identifies each linkage between associated rows of data in separate forms consistently with Fig. 1, the serial number of the destination form is recorded on the source form. To enable cleaning of data for this unique identifier, the *serial* and *row* numbers of the relevant data row from the source form also are recorded on the destination form to provide an alternative identifier. Appropriate sample storage involves not only clear labelling of each sample, but also a record of where, when, how and by whom the samples were stored. Therefore, the long-term archiving of samples is recorded using the ST sample storage forms, allowing samples to be located easily at a later date, based on the system of sample labelling described below.

### Sample labelling and storage

**Fig. 3:**
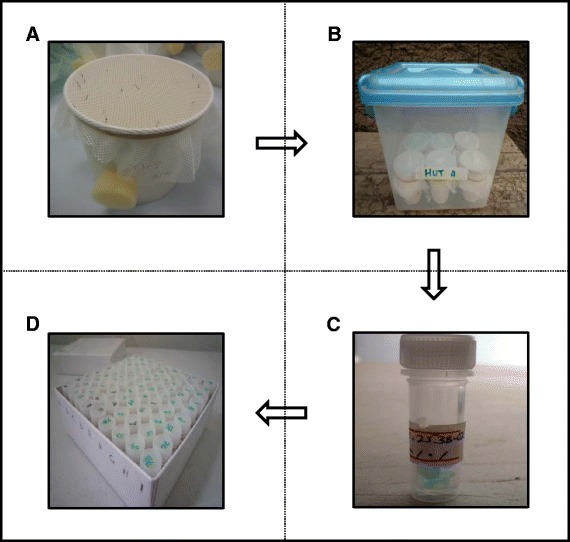
Systematic labelling of collection cups and mosquito samples. The cups containing each mosquito collection are labelled clearly and meaningfully to uniquely identify each cup at each experimental time point (e.g., household number, time and trap type) (**a**) and then placed in a container (**b**). Mosquitoes are sorted and placed in a tube which is identified uniquely by combining sorting form type, form serial number, form row, body form (to distinguish intact from carcass samples) sample type (to distinguish individuals, batches and pools) and sample identifier (to distinguish distinct samples of a single type) (**c**). Labelled tubes with samples are then placed inside a storage box along with the SO form (**d**)

Before each experiment begins, the collection cups used to contain each mosquito collection are labelled clearly and meaningfully. The label should include all-important information that uniquely identify each cup at each experimental time point (e.g., *household number, time and trap type*) for use by the researchers when conducting the experiment. For some complex experiments, large numbers of collections (>100 in some experimental hut studies we have implemented) will need to be handled during each experimental unit at a given time (e.g., replicate night). To maintain order during such large experiments, we recommend grouping the collection cups by information-rich data (i.e., information that can be used to identify each cup) in separate holding boxes, ideally with each box corresponding to one experimental design form. In addition to the information-rich label, the cup should also be labelled with the corresponding *serial number* and f*orm row* identifiers from the form. The researcher should use the form *serial* and *row* numbers to sort the collections cups sequentially, thus enforcing a structured order to the data record. Although, the form *serial* and *row* numbers are sufficient to uniquely identify collection and derived samples, information-rich details that have intuitive meaning to field personnel also should be included so they can readily cross-check and correct errors in the sample labels or corresponding data on ED and SS forms (Fig. 3).

Collections are usually sorted into several derived samples, some of which may be split into sub-samples from a single sort category for further processing and storage. Furthermore, these samples and subsamples may be processed for further observations (SO form), so it is essential to trace the exact identity and origins of each individual sample. Therefore, each sample of intact insects is identified uniquely by combining *form type, form serial number, form row, sample type* (to distinguish individuals, batches and pools) and *sample identifier* (to distinguish distinct samples of a single type) (Fig. 3, c) attributes to generate a primary key which takes the user to the exact place on the form where the sample was created. However, one sample of intact insects may be split into multiple body components during the observation processes, such as dissection or preparation for molecular analysis, e.g., the head and legs may be stored and processed separately, so the *body form* attribute also is recorded on both the SO form and the sample label to distinguish these sub-samples of the insect carcass. From here, the user can link to all recorded experimental design, sorting or observation attributes for that sample. An alternative key for uniquely identifying samples may be recorded at the user’s discretion as a single *sample label code* attribute on both the paper-based form and the sample label. The sample label code may take the form of any unique code the user chooses, generated by whichever automated or manual system is available. However, we suggest using the ‘current date’ just the first six digits (ddmmyy), to distinguish one sample from another, three digits can be added after the current date starting from 001 onwards depending on the number of samples needed to be labelled for that particular day. For example, if one has three SS1 rows (e.g., 4, 10, and 17) with data, where the sorting was done in Feb 20, 2013 the SLCs are 200213001, 200213002, and 200213003 respectively, while if the sorting was done in Oct 4, 2013 then SLCs are 041013001, 041013002, and 041013003 respectively. This approach is preferred because it is an easy one to implement and it does not require prior knowledge of the label code used.

The sample storage box record form (ST) is uniquely identified by a *serial number*, which records sample storage information for each storage box. The box contain labelled samples and filled-in SO form such as *Box & form serial number (*to distinguish distinct storage boxes from the same or different experiments and/or projects), *number of samples, storage temperature, crate/freezer/fridge number,* and *rack or carton number*.

Data collected using the forms described above, once linked and stored in a given relational database, may be linked easily with environmental or any other demographic data for a given geographic area. This is possible because the ED form captures the unique house number where available. For example, using a unique house number recorded using ED1, data from the demographic surveillance system (DSS), which also contain a unique house number for the same location, can be linked together with mosquito entomology data.

## Results

Data from the majority of mosquito studies conformed to the proposed generic schema with data collection forms recording the experimental design, sorting of collections, details of sample pooling or subdivision, and additional observations. Such mosquito studies include, but are not limited to, [34–45]: Survey of indoor human exposure to malaria transmission, survey of immature mosquitoes from natural field habitats, experimental hut assays of adult mosquito susceptibility to insecticides, insecticide susceptibility bioassay under laboratory condition and on-going insecticide resistance studies based on wild mosqutoes in Zambia’ as illustrated using the following selected examples. These examples are based on the studies that were conducted by either authors or non-authors who are research scientists at Ifakara Health Institute, Tanzania, Zanzibar Malaria Control Program, and Zambia Malaria Control Center.

### Illustrative examples

Figure 1 defines the direction and function of the relationships between each experimental stage. Clearly, there are very large number of possible experimental designs that could be followed, so selected examples are provided to illustrate how the generic forms and underlying schema were applied to achieve specific experimental objectives. The step-by-step procedures involved in the four experiments described below, plus three other experiments are given in the appendix, show how data collection forms were filled with data for specific attributes [see Additional file 3]. Once an understanding is gained of how specific experiments can be nested within the schema, and specific form designs are selected from each form category, it is relatively straightforward to adopt this system for a wide diversity of other experimental designs as long as the experiment follows some or all of the commonly used entomological experimental procedures (i.e., *experimental design*, followed by *sample collection, sorting, constitution, and observation*).

#### Example 1: A demographically representative survey of indoor human exposure to malaria transmission

This longitudinal survey of a mosquito population was designed and implemented to evaluate the quantitative relationships between mosquito ecology, coverage of long-lasting insecticidal nets (LLINs) as a vector control measure, and entomological indicators of malaria transmission intensity [46, 47]. The intensity of human exposure to malaria transmission was estimated as the entomological inoculation rate (number of infectious bites by sporozoite-infected mosquitoes per person per year) [48, 49].

In Africa generally [50], and this rural Tanzanian study site specifically [51], the main malaria vectors primarily feed upon humans while they are asleep indoors, so CDC light traps placed beside bed nets occupied by people are a reliable, widely-practiced means to collect them. After each night of collection in houses selected at random from a demographic sampling frame consisting of a village household list (recorded using ED1), the mosquitoes caught in each trap were placed in labelled cups, killed, sorted and counted to enumerate each mosquito category and yield defined samples (SS1). Samples of individual mosquitoes were observed in the field with a microscope to measure wing length and to determine gonotrophic age following ovarian dissection [30] (SO2). Then the samples were transferred to a separate laboratory team who determined sporozoite infection status for each specimen using enzyme-linked immune-absorbent assay (ELISA) [28], and sibling species identity of the *An. gambiae* complex specimens were determined using PCR [26] (SO1). The DNA and carcasses of the mosquito samples were archived for long-term sample storage, with their placement in 81-cell storage boxes and location of boxes in the laboratory recorded using the *box record* form (ST).

#### Example 2: Survey of immature mosquitoes from natural field habitats

It is also common to collect immature mosquitoes in their natural aquatic habitats as part of field surveys or experiments, similarly to the way adults were surveyed in example 1. In this example, routine surveillance of larval habitats in urban Dar es Salaam in Tanzania was conducted to monitor effectiveness of a city-level larval source management program and to identify strengths, weaknesses, and opportunities for improvement in the routine internal monitoring systems of that programme [39, 40, 52]. The details of where, when and how each collection of aquatic stage mosquitoes was obtained by dipping in carefully catalogued habitats in well-mapped enumeration areas [53–55] were recorded *as date*, *enumeration area*, *compound*/*plot*, *habitat number*, *habitat type*, collection *method* and *number of dips* attributes in a single row of an experimental design form (ED2), based on prototypes [46] that have been refined through practical use over several years. After collection, the larvae were sorted into predefined categories based on *taxon* (*Anopheles* spp.*, Culex* spp. *Aedes* spp,) and *body form* (egg, early stage larva (instars 1 & 2), late stage larva (instars 3 & 4)), attributes that are pre-filled into the sort form for field collections of immature stages (SS2). In this case, all collected immature mosquitoes were discarded, but the sort category, constituent number of specimens, and identity attributes of samples retained for experiments, observations and storage may be readily recorded in SS2, which links to optional additional sample observation (SO1, SO3), constitution (SC1) and storage form (ST) just as described for adult mosquitoes and associated sort forms (SS1/SS3).

#### Example 3: Experimental hut assays of adult mosquito susceptibility to insecticides

This example illustrates the design of a small-scale field evaluation of the efficacy of several combinations of alternative LLIN and indoor residual spray (IRS) products against natural populations of mosquitoes in Zambia, under realistic but well-controlled field conditions, using experimental huts [25, 56–58]. The procedures applied to this experiment are essentially identical to published studies from Tanzania, in which several alternative vector control product combinations were assessed, comparing their deterrency, mortality, blood-feeding inhibition and induced exophily (house exit) [9], all of which were used as input parameters for simulations of expected community-level impact [10].

*Date, enumeration area (village), method, indoor/outdoor, start time, finish time, round, house/hut, volunteer initials, treatment (LLIN or untreated net) and experimental day* attributes for each of several separate collections from within each hut was recorded on a separate line of an experimental design form (ED1). To assess delayed mortality amongst the captured mosquitoes, all live mosquitoes from each collection were then held for 24 h in a separate holding container with a supply of glucose solution in a field insectary. After the holding period, each collection of mosquitoes was sorted into subgroups using the categorical attributes *dead*, *taxon*, *sex* and *abdominal status*. The number in each subgroup was counted, and the derived samples of mosquitoes were placed in labelled storage tubes, all details of which were recorded in a single sort form (SS3) for each collection. These samples then were passed to a separate laboratory team who determined sporozoite infection status by ELISA [59] and sibling species identity by PCR [26] and recorded these attributes on a sample observation form (SO1). The remaining carcasses of the mosquito samples then were archived for long-term sample storage (ST).

#### Example 4: Insecticide susceptibility bioassay under laboratory conditions

Before insecticides can be used for controlling wild vector populations in the field, it is essential to determine the optimal formulation and dosage to maximize efficacy and residual activity though laboratory experiments [25]. In this example, the mortality response of adult mosquitoes when exposed to entomopathogenic fungi was tested under insectary conditions [41]. The experiment was conducted by creating multiple collections from an insectary colony of *An. gambiae*, each of which is a single batch (usually >20) of live mosquitoes, each of which is assigned to one experimental replicate for which the source of mosquitoes (*colony code*), *sex and abdominal status*, *age*, *number of mosquitoes*, *start date*, *treatment* and *replicate* attributes were recorded in one row of an experimental design form (ED2). Each batch was treated in the same manner, except that mosquitoes in different batches were exposed to different experimental treatments, specifically a range of concentrations of fungal conidia. After pre-defined holding periods at intervals of 24 h, the mosquitoes were sorted on the basis of being dead on that experimental day or still alive at the end of the experiment. In this example, the duration of each sequential holding period defined by the experimental design was recorded in a sort form for batches or pools (SS3) as the *finish date* for each holding period, but this also can be more directly recorded as the *holding period* attribute. The number of mosquitoes in each category of *holding period* and *survival status* was observed by counting from each collection/experimental unit, and the number in each category was recorded on one SS3 form. In this example using insectary mosquitoes from a known, presumably homogenous genetic and environmental background, no samples were retained for storage or further observation.

Therefore, although, the four examples described above are different and from diversity study areas they all conformed to the generic schema and data could be collected using the paper-based data collection forms with standardized data formats.

### End-user uptake

The generic schema and forms initially were developed and piloted at the Ifakara Health Institute (IHI) in Tanzania in 2008 and subsequently evolved through interaction with end-users adopting it for specific projects. The subsequent demand for these generic, broadly applicable schema and data collection tools are demonstrated by growth of the user base over the following 5 years to encompass 20 experiments, 8 projects, and 8 project investigators working on a wide range of vector ecology and control issues at IHI and the National Institute for Medical Research (NIMR) as well as collaborating national malaria control programmes in mainland Tanzania, Zanzibar, and Zambia, resulting in 11 peer-reviewed publications [34–45].

## Discussions

The generic schema described here captures the complexities of diverse mosquito-based experiments into a common, consistent, simplified structure that can be conceptualized by most mosquito entomologists. The data collection forms developed from the generic schema provide a framework for the processing and handling of both samples and data. It is essential to record not only the processing of the samples after collection, but also the specific experimental design and methods implemented because results only have context with regard to the way the samples were collected and observations were made.

This study has several limitations including 1) Inability to pre-define all possible experimental variables; 2) Users’ reluctant to adapt to a new system; 3) Some technical issues such as the quantity of data collection forms that may be required during an experiment. We discuss each in turn.

Fortunately, even though we cannot predefine all the variables, the proposed generic schema provide a framework that can be used to add any new defined variable as long as the user knows the category (i.e., experimental design or sample collection or sample sorting, or sample constitution, or sample observation) in which a new variable belongs. Also, columns for experiment-specific attributes, which are not captured by the generic options, also allow the user to define codes for these additional variables. Blank columns in data collection forms are also available for new user-defined attributes.

We understand that in most circumstances, users are always reluctant to adapt to the new systems. We have also observed this to be the challenge when we first introduced the generic schema and data collection forms to different project investigators. We were able to overcome the challenge by explaining the advantages of the proposed system such as reducing the time required to redesign data collection forms for each new experiment, an easy approach to link field and laboratory data, and abilities to share data with standardized formats from multiple study sites leading to increase in research outputs. We also provided required training to ensure that users are comfortable and understand how generic schema and data collection forms can be used. In addition, another caveat might be the quantity of data collections forms that need to be printed for a specific experiment. While testing the proposed data collection forms, some users noticed that they needed to print more forms than they would normally print if they had designed their own forms to record only intended variables for their specific experiment. This may be the case in some experiments but it is necessary that the proposed forms are printed as needed to take full advantage of the proposed system for improved data quality ensuring that data collected is clear and unambiguous. In addition, users may opt to shade the columns with variables that will not be recorded in the forms during their specific experiment to make it easier for data recording. The users opting to use electronic-based data collection forms can customize the forms by selecting only required variables required for their specific experiment.

Electronic data collection devices, such as PDAs or mobile phones, provide many advantages over paper forms to the user. However, these also often require a highly specialised and customised user interface that usually is tailored to the specific collection methods and/or experimental tasks [60]. Designing and supporting electronic user interfaces is a non-trivial task, so this flexible paper-based system may be most useful to under-resourced medical entomology groups in developing countries lacking sufficient access to specialist software or expert support to develop tailor applications to each individual studies.

The broad applicability of the data collection forms enables consistent application of this schema, as well as robust standardization of attribute definitions, both within and between experiments. Furthermore, these forms eliminate the need to redesign forms and databases for each experiment – a laborious and often error-prone task which can be prohibitively resource-intensive, especially when multiple diverse, sometimes iteratively-designed, experiments are conducted over short periods by large research groups, consortia or communities. The data collected will contain standardized data formats allowing a wide research community to share data and to address questions beyond a given project’s specific objectives hence increasing research output.

The data collected using proposed forms can be entered and linked with any relational database depending on user’s choice. Our next step is to develop electronic version of the proposed data collection forms and make it available to users who will opt to use electronic devices with no in-house informatics experts to develop the software. In addition, we are developing a database web-based application based on the proposed generic schema that can be used to store, link, share authorized data from multiple experiments, projects, and study sites and generate summarized reports. Such repository will provide the malaria research community with quality data with standardized formats from multiple study sites that can be used to address several scientific questions from household to national to regional level. Data with finer scale (i.e., collected at household level) with information such as where, how, and when the malaria vectors were collected, their behavioural and physiological characteristics, as well as their transmission activities. We will ensure that such system will easily be linked to complement international repositories such as MAP and VectorBase.

## Conclusion

We have designed generic schema that can be used to develop paper or electronic based data collection forms depending on the availability of resources. We have developed paper-based data collection forms that can be used to collect data from majority of entomological studies across multiple study areas using standardized data formats. Data recorded on these forms with standardized formats can be entered and linked with any relational database software. These informatics tools are highly recommended because they ensure that entomologists save time, improve data quality, and data collected and shared across multiple studies is clear, unambiguous, and in standardized formats hence increasing research outputs.

## Additional files

Additional file 1: The data collection forms. This excel document allows users to easily edit and print the data collection forms. The master dictionary is also included with the data collection forms. (XLSX 143 kb) Additional file 2: Standard operating protocol for application of the forms. This document provides semantic details for each data collection form. (DOCX 27 kb) Additional file 3: Examples from various experiments to show how the data collection forms can be used. This document provides step-by-step descriptions of exactly how the four examples (with addition of 3 other examples) were executed using the data collection forms. (DOCX 15304 kb)
