# Supplementary material for: A generic schema and data collection forms applicable to diverse entomological studies of mosquitoes
Source: Source Code Biol Med. 2016 Mar 28;11:4. doi: 10.1186/s13029-016-0050-1 (PMC4809029; doi:10.1186/s13029-016-0050-1)
Supplement: Additional file 2: — Standard operating protocol for application of the forms. This document provides semantic details for each data collection form. (DOCX 27 kb) [file 13029_2016_50_MOESM2_ESM.docx]

**Version 1.1**

**Supplementary Online Material – File 2**

A generic schema and standardized data collection forms that are applicable to diverse entomological studies of mosquitoes

**Definitions and Standard Operating Procedures**

Samson Kiware^1,2,*^, Tanya L Russell^1,3^, Zacharia J Mtema^1^, Alpha D Malishee^1^, Prosper Chaki^1^, Dickson Lwetoijera^1,4^, Javan Chanda^5^, Dingani Chinula^5^, Silas Majambere^1,4^, John E Gimnig^6^, Thomas A Smith^7^, and Gerry F Killeen^1,4^

^1^Environmental Health and Ecological Sciences Thematic Group, Ifakara Health Institute, P.O. Box 53, Ifakara, Tanzania

^2^Department of Mathematics, Statistics and Computer Science, Marquette University, Milwaukee, WI 53201-1881, USA

^3^Pacific Malaria Initiative Support Centre, School of Population Health, University of Queensland, Brisbane, 4006, Australia

^4^Vector Biology Department, Liverpool School of Tropical Medicine, Pembroke Place, Liverpool, L3 5QA, United Kingdom

^5^National Malaria Control Centre, Lusaka, Zambia

^6^Division of Parasitic Diseases, Centers for Disease Control and Prevention, Atlanta, GA, USA

^7^Department of Public Health and Epidemiology, Swiss Tropical Institute, Socinstrasse 57, Basel, CH 4002, Switzerland

**An Overview of the Data Collection Forms**

There are six categories of the data collection forms, each designed for specific functions:

1. *Informed consent record:* Records the written informed consent forms that are completed by experiment volunteers;
2. *Experimental design:* Records the design of an experiment;
3. *Sample sorting forms*: Records the process where a sample of > 1 mosquitoes (either collection, batch or pool) is broken into subgroups using pre-defined categories;
4. *Sample observation*: Records the results of a direct scientific observation made of an individual, batch or pool;
5. *Sample constitution:* Creates new samples of clearly defined origin from multiple contributing samples; and
6. *Sample Storage*: Records the long-term storage, location, and conditions for samples in the laboratory.

Within each category, there are up to 3 alternative form designs to encompass the various experimental procedures that may be followed (TABLE S2.1).

Table S2 1. Details of the six categories of the data collection forms

| **Category** | **Unique Identifier** | **Form** | **Description** |
| --- | --- | --- | --- |
| **Informed consent record** | IC1 | Informed consent record | Details of written informed consent forms |
| **Experimental design** | ED1 | Field collections | Records the design of experiments collecting mosquitoes in the field |
|  | ED2 | Batch and/or pool experimental assay | Records the design of experiments using colony mosquitoes or pre-existing batches or pools |
| **Sample sorting** | SS1 | Adult field collection | Records the process by which a field collection of mosquitoes is sorted into pre-defined subgroups of taxon, sex and abdominal status |
|  | SS2 | Immature field collection | Records the process where a field collection of immature mosquitoes is sorted into each specified combination of taxon and body-part |
|  | SS3 | Batch and/or pool experimental assay | Records the process where a batch and/or pool of mosquitoes is experimentally sorted into pre-defined categories reflecting specific behavioural physiological or mortality responses |
| **Sample observation** | SO1 | Laboratory analysis | Scientific observations made using molecular analysis of mosquito samples |
|  | SO2 | Dissection and wing length | Scientific observations made to measure the parity status (females only) and wing length of dead individual mosquitoes |
|  | SO3 | Individual experimental assay | Various scientific observations of individual mosquitoes made in the field or entomology laboratory |
| **Sample constitution** | SC1 | Pools | Records the process where multiple samples are pooled into a single sample |
| **Sample storage** | ST1 | Box contents | Records the contents of an 81-cell sample storage box |
|  | ST2 | Box record | Records the long-term storage location of sample boxes in the laboratory |

**Informed Consent Record Form (IC1)**

Before commencing an experiment the scientist must ensure that appropriate ethical approval has been granted, and that the volunteers are willing to participate. Volunteers can give written approval by completing an informed consent form, and a record of the forms can be created using the IC1 form. Appropriate storage and recording of informed consent forms allows the scientist and auditors to keep track of this essential process, especially if hundreds of volunteers have been enrolled. The informed consent forms may need to be accessed during the follow-up stages of a trial if scientists need to confirm that the process was completed or if scientists want to know if a specific household has been enrolled in the trial. Volunteer approval is confidential, and these forms should be treated with due care, kept in a locked filing cabinet and all electronic records password protected. Each informed consent form (completed by one volunteer) is represented by one row in the IC1 form. Each informed consent form record can be uniquely identified (primary key) by serial number (SEN), form row (FR) project code (PC), experiment number (EN), and site (SI) on the IC1 form.

**Experimental Design (ED1 or ED2)**

This category contains two types of experimental design data collection forms for (1) adult field collection (ED1), and 2) batch and/or pool experimental assay (ED2).

**Experimental design form: Adult field collections (ED1)**

The form records the “where”, “when”, and “how” details of one or more distinct collections of mosquitoes from a field population are obtained each from a single trapping or sampling effort. Each collection is represented by one row in the form. There are many attributes that can be recorded about each collection. Before the experiment commences, the user has the option to designate which optional attributes should be recorded for each specific project and to pre-fill the experimental design into the forms before printing. Each population collection can be uniquely identified (primary key) by site (SI), project code (PC), experiment number (EN), serial number (SEN), and the form row (FR).

**Experimental design form: Batch and/or pool experimental assay (ED2)**

This form records the experimental design that was followed when conducting an experiment using batch and/or pooled samples of mosquitoes obtained from an insectary colony or progeny rearing. Each form record can be uniquely identified by project code (PC), experiment number (EN), site (SI), serial number (SEN), and form row (FR).

**Sample Sorting (SS1, SS2, or SS3)**

This category contains three sample sorting data collections forms 1) adult field collections (SS1), 2) Immature field collections (SS2), and 3) batch and/or pool experimental assay (SS3).

**Sample sorting form: Adult field collections (SS1)**

This form records sorted adult mosquitoes after a sorting process by which a collection of adult mosquitoes is sorted into each possible combination of ‘*Taxon*’ and ‘*Sex and Abdominal Status*’. The classification outcomes of sorting, counting, and processing samples from each collection is recorded using one destination SS1 form that corresponds to one line in the ED1 form. After the collection has been sorted the mosquitoes can be either processed as samples (individuals or batches) or discarded and the details are recorded on the right hand part of the form. To ensure storage conditions that will enable a long-term preservation of the mosquitoes and DNA, it is recommended that batches should contain no more than 10 mosquitoes. If the user is planning to process the samples individually at a later date, it is recommended that the samples are stored as individuals. Each form record is uniquely identified (primary key) by the form serial number (SEN), form row (FR), sample type (ST), and sample ID (SID) or by the sample label code (SLC).

**Sample sorting form: Immature field collections (SS2)**

The sample sorting form for immature field collections (SS2) records a collection of immature mosquitoes. If that collection was obtained by sampling natural population of immature mosquitoes, all relevant details of that collection maybe found in a single line of an ED1 source form for each classification system and outcomes of sorting, counting, and processing samples. However, if the collection used was obtained from insectary-reared immature stages, these details are described in an ED2 source form. After the collection has been sorted the mosquitoes can be either processed as samples (individuals or batches) or discarded. This process is recorded on the right hand part of the form. Each form record is uniquely identified (primary key) by the form serial number (SEN), form row (FR), sample type (ST), and sample ID (SID) or by the sample label code (SLC).

**Sample Labelling**

At this stage, it is important to explain how to label collection cups and samples for storage so that they can be traced.

**Labelling collection cups during experimental procedures**

Before set of experimental or survey collection is made, as recorded in an ED form, the cups that will be used to contain mosquitoes after they are collected need to be labelled clearly, uniquely, and meaningfully. The label needs to be meaningful and contain all features of the experimental design that uniquely identify each cup on each day of collection in a format that makes intuitive sense. In addition to this, the cup also should contain the corresponding serial number (SEN) and form row of the corresponding row on an experimental design (ED) form. When sorting the cups the following morning, the staff executing the experiment or survey should use the serial number and form row to sort the cups in sequential order. Remember that if the mosquitoes are to be held for survival analysis to use cups with rough inner surfaces in which they can comfortably grip and rest on the walls. For example, a cup may be labelled as follows:

House: 4

Time: 19 – 20

Trap: HLC

Date: 14/11/11

ED1 FR: 1

**Labelling and storing samples**

To enable each sample to be traced, it is assigned two independent uniquely identifying label codes – one as primary and another one as an optional alternative key. Each sample needs to be labelled clearly to allow the scientist to link the sample with recorded data at a later date. A small label should be stuck onto the outside of the tube and contain the following information – first and second lines (primary key) and third line (alternative key):

FT-SEN-FR

BF-ST-SID

SLC

First Line:

Second Line:

Third Line:

Where**:** FT = Form type, SEN = Form serial number, FR = Form row, BF = Body Form, ST = Sample Type, SID = Sample ID, and SLC = Sample Label Code.

The primary key used to identify each sample within each experiment is ‘Form Serial Number.’ + ‘Form Row’+ ‘Sample Type’ + ‘Sample ID’. This label will take the user to the exact place on the form where the sample was created. From here the user can link with the other experimental data, such as experimental design or scientific observations. The alternative key is also recorded on both the paper-based form and the sample label and is a uniquely generated: ‘Sample Label Code (SLC)’. The Sample Label Code is shorter and simpler and may be used instead to uniquely identify a sample. Researcher may choose any approach in generating a unique SLC. However, it is recommended using the ‘current date’ just the first six digits (ddmmyy) followed by another three digits starting from 001 on wards to differentiate one sample from another.

**Sample Observation Forms (SO1, SO2, or SO3)**

This category contains three sample observation forms 1) laboratory analysis (SO1), 2) dissection and wing length observation (SO2), and 3) individual experimental assay (SO3).

**Sample observation form: Laboratory analysis (SO1)**

The sample observation form for laboratory analysis records scientific observations made using molecular analysis of individual or batch samples of mosquito. The common molecular analyses on the form are: *species identification, KDR resistance, Plasmodium infection status* and *bloodmeal host identification*. The research can select which of the molecular analyses are required for each mosquito by marking an ‘X’ in the *required* (RQ) box. The result is then written in the corresponding *results* (RS) box. Each sample submitted to the laboratory for analysis is represented by one line of a laboratory analysis sample observation form (SO1), and it is uniquely identified by form serial number (SEN) and form row (FR) or alternatively by using the source sample label code (SSLC). Laboratory analysis and storage service cost agreement information is captured by the fourth page of SO1 form. For example, the form details the service costs of the requested laboratory analyses as used as an invoice support document in institutional admin system at Ifakara Health Institute.

**Sample observation form: Dissection and wing (SO2)**

The sample observation form for dissection and wing observations (SO2) records scientific observations made to determine the parity status (females only) as well as wing length of dead individual mosquitoes. The user can select which of the observations are required for each mosquito by marking an ‘X’ under the observation heading. Each observed and processed sample is uniquely identified by form serial number (SEN) and form row (FR) or alternatively by using the source sample label code (SSLC).

**Sample storage (ST1 and ST2)**

This category contains two storage forms 1) box content and 2) box records.

**Sample storage: box contents form (ST1)**

This form records the contents of an 81 cell sample storage box with rows (A to I) and columns (1 to 9). The mosquito samples should usually be stored in 1.5 or 2 ml micro-centrifuge tubes containing a small amount of silica gel separated from the sample by a thin layer of cotton wool. Each sample can be uniquely identified by the form serial number (SEN) and form row (FR).

**Sample storage: box record form (ST2)**

The form records the long-term storage of sample boxes in the laboratory. There is a provision for the scientists to indicate under what conditions are samples need to be stored. Each individual sample storage box can be uniquely identified by the form and box serial number (SEN).
