# Supplementary material for: A generic schema and data collection forms applicable to diverse entomological studies of mosquitoes
Source: Source Code Biol Med. 2016 Mar 28;11:4. doi: 10.1186/s13029-016-0050-1 (PMC4809029; doi:10.1186/s13029-016-0050-1)
Supplement: Additional file 3: — Examples from various experiments to show how the data collection forms can be used. This document provides step-by-step descriptions of exactly how the four examples (with addition of 3 other examples) were executed using the data collection forms. (DOCX 15304 kb) [file 13029_2016_50_MOESM3_ESM.docx]

**Additional file 3**

A generic schema and standardized data collection forms that are applicable to diverse entomological studies of mosquitoes

**Examples from various experiments to show how the data collection forms can be used**

**Example 1: A demographically representative survey of indoor human exposure to malaria transmission**

The steps involved in the experiment were as follows:

1. **Collect mosquitoes**: One collection is the same as one trapping effort and mosquitoes are placed into one collection cup. Recorded using one line on the experimental design form (ED1). Data was recorded for the following collection attributes: d*ate of collection, enumeration area (village), household, method, indoor/outdoor, start time, finish time* and *experimental day (*Figure S2 1). A blank cell indicates that a particular variable was not recorded during the experiment. Users may opt to shade the columns that will not be used during an experiment. This is the case in all the data collection forms.


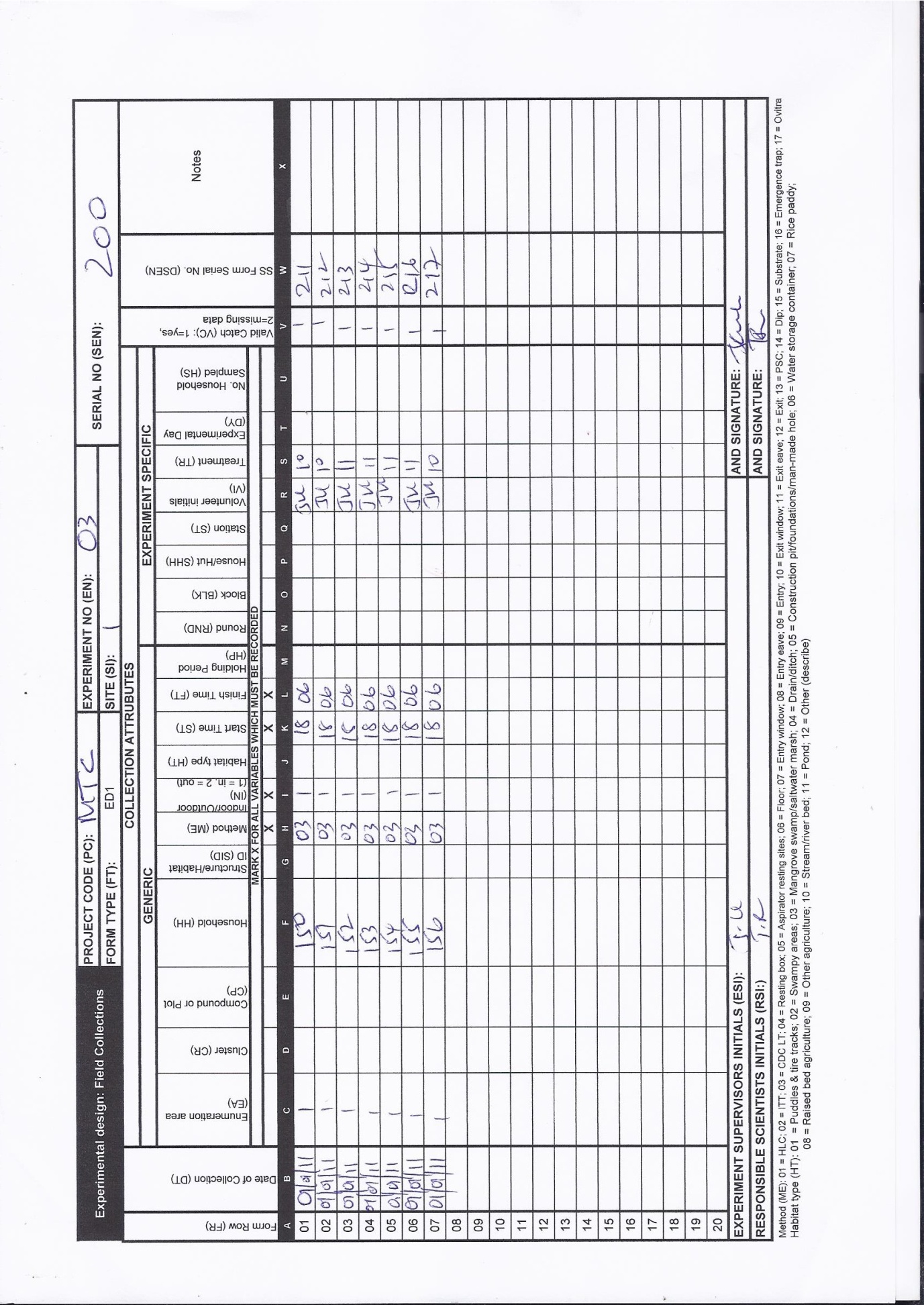


Figure S2 1: The ED1 form where the required attributes are filled-in for a demographically representative survey of indoor human exposure to malaria transmission experiment. In this particular example Site 1 represents Ifakara and Enumaration area represents Lupiro village (i.e., one of the village under Ifakara study site). Treatment =10 (untreated nets) and (treatment =11 (long lasting insecticide nets)

1. **Sort collection:** Each collection was sorted into pre-defined subgroups of *taxon, sex* and *abdominal status*. Both these sample sorting attributes are categorical, as follows:
   1. Taxon: *A. gambiae s.l., A. funestus,* culex spp., Aedes spp., Mansonia spp.
   2. Sex and abdominal status: *total male*, *unfed, part fed, fed gravid/semigravid, total female*

The number in each subgroup is counted, and the data from each collection was recorded using one sample sorting form (SS1). As an example, two SS1 forms are filled-in corresponding to the first (Figure S2 2) and third rows of ED1 respectively.

Figure S2 2: The SS1 containing sample sorting information (the number caught for the three species, their sample type and the number discarder for the first row of ED1 from Figure S2 1.


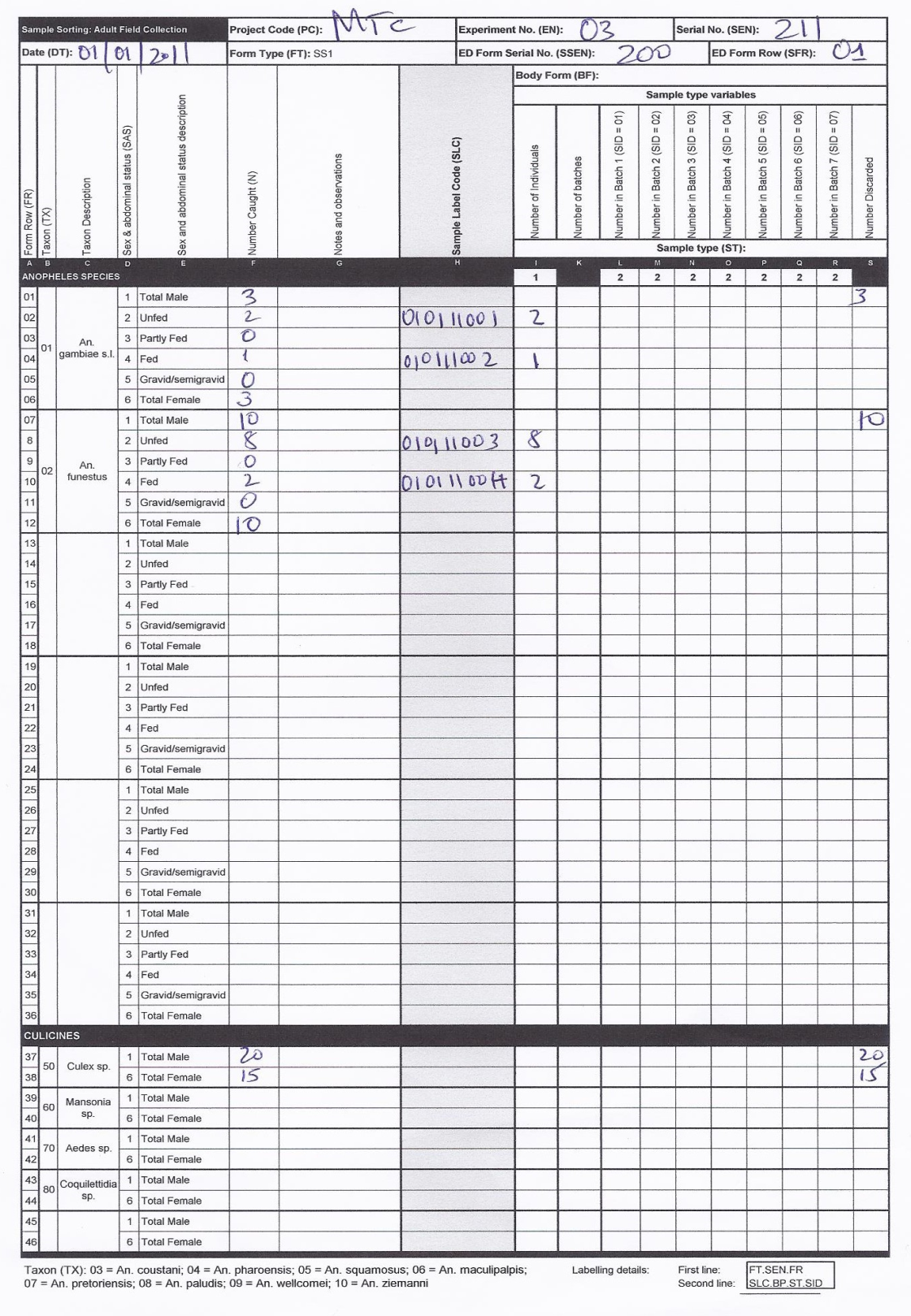


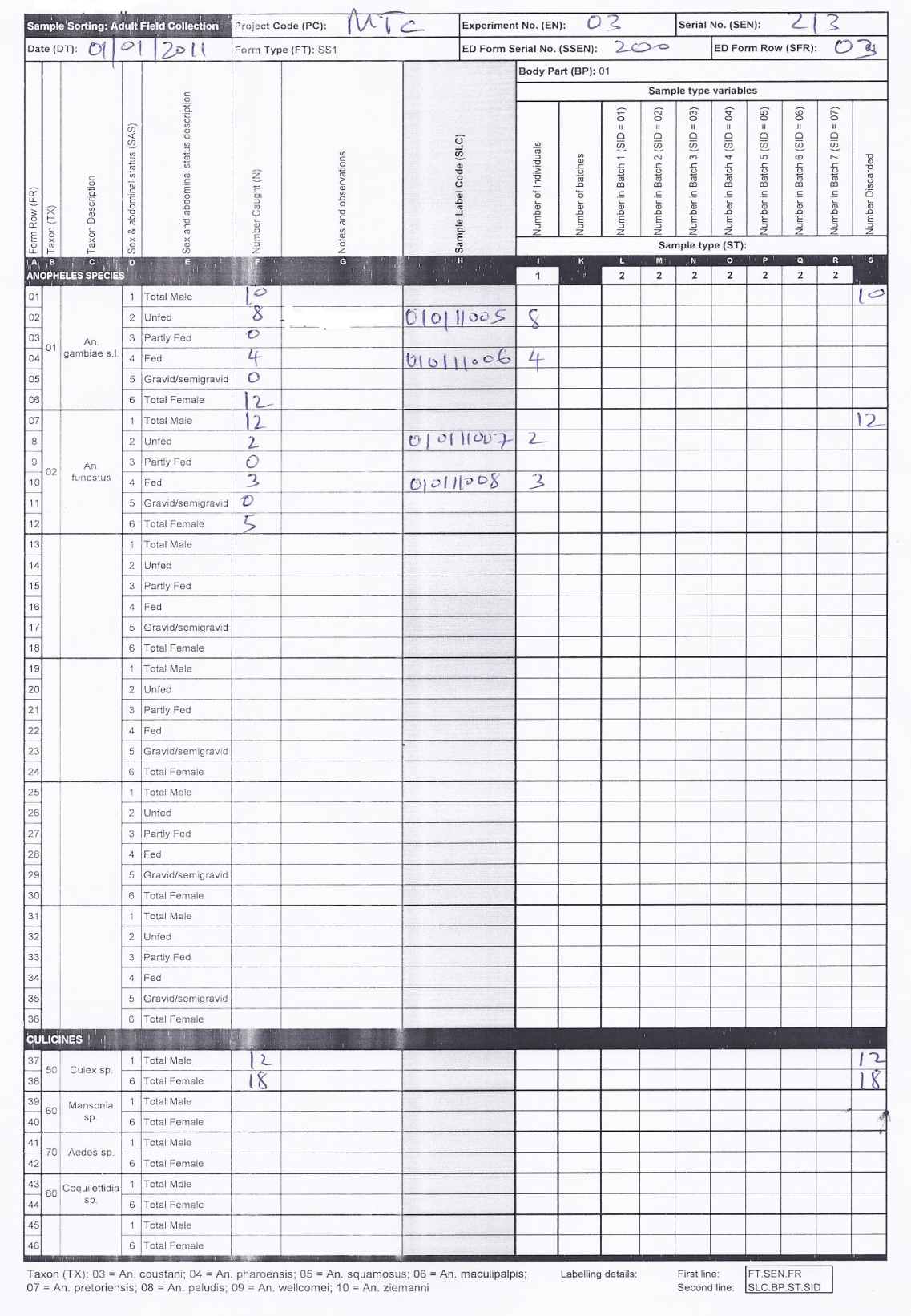


Figure S2 3: The SS1 containing sample sorting information (the number caught for the three species, their sample type and the number discarder for the third row of ED1 from from Figure S2 1.

1. **Create samples:** During the sorting process, samples of individual (n = 1) or batches (n ≥ 2) of mosquitoes may be created, labelled and stored. Due to the large number of female mosquitoes caught per trapping effort, separate random sub-samples, each averaging approximately 10% of the total in each trap, were constituted for storage and further subsequent observations. In cases where the catch was less than 10 mosquitoes, all specimens were retained as individual samples. Individual samples were created for further observations in step 4 for collection that yielded > 10 mosquitoes in a single, Anopheles category of *taxon* and *sex and abdominal status*. The individual samples was recorded on the corresponding sample sorting form (SS1) used in step 2 on the far right-hand panel. Each individual sample is labelled using three lines where the first one contains form type, *form serial number, and form row, the second one contains body form, sample type,* and *sample ID*, the third line contains the *sample label code* which is optional.


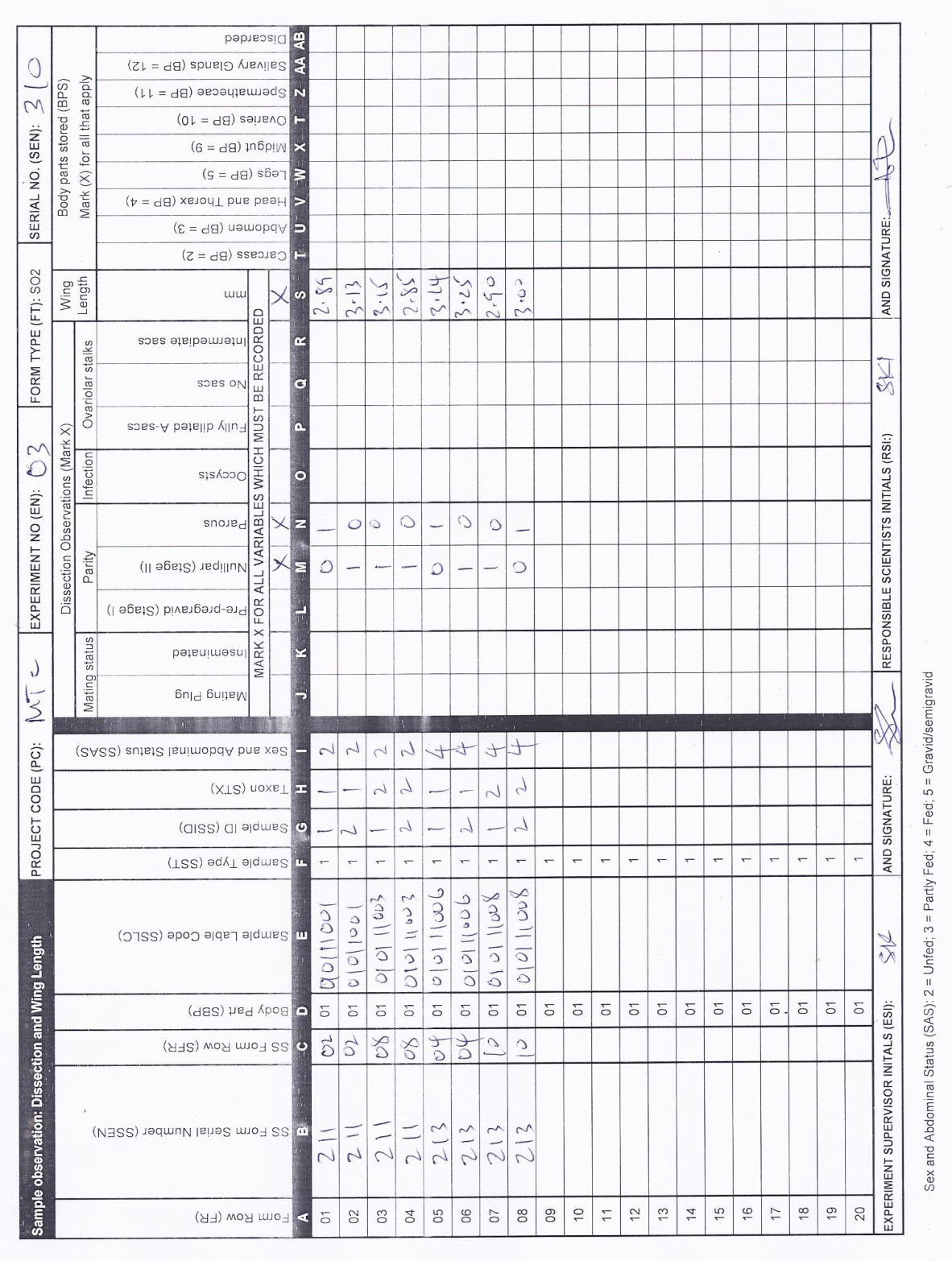


Figure S2 4: The SO2 form recording dissection and wing length information for selected samples coming from SS1 with serial numbers 211 and 213 just as an example.

1. **Sample observation:** The *parity status* and *wing length* of the individual mosquito samples was measured and recorded on a sample observation form for dissections and wing length measurements (SO2) (Figure S2 4).
2. **Sample observation:** At a later date, the *infection status* of each sub-sampled mosquito was observed using ELISA, and the *sibling species identity* of individual samples was determined with PCR and recorded on a sample observation form for laboratory assays (SO1) (Figure S2 5).

**
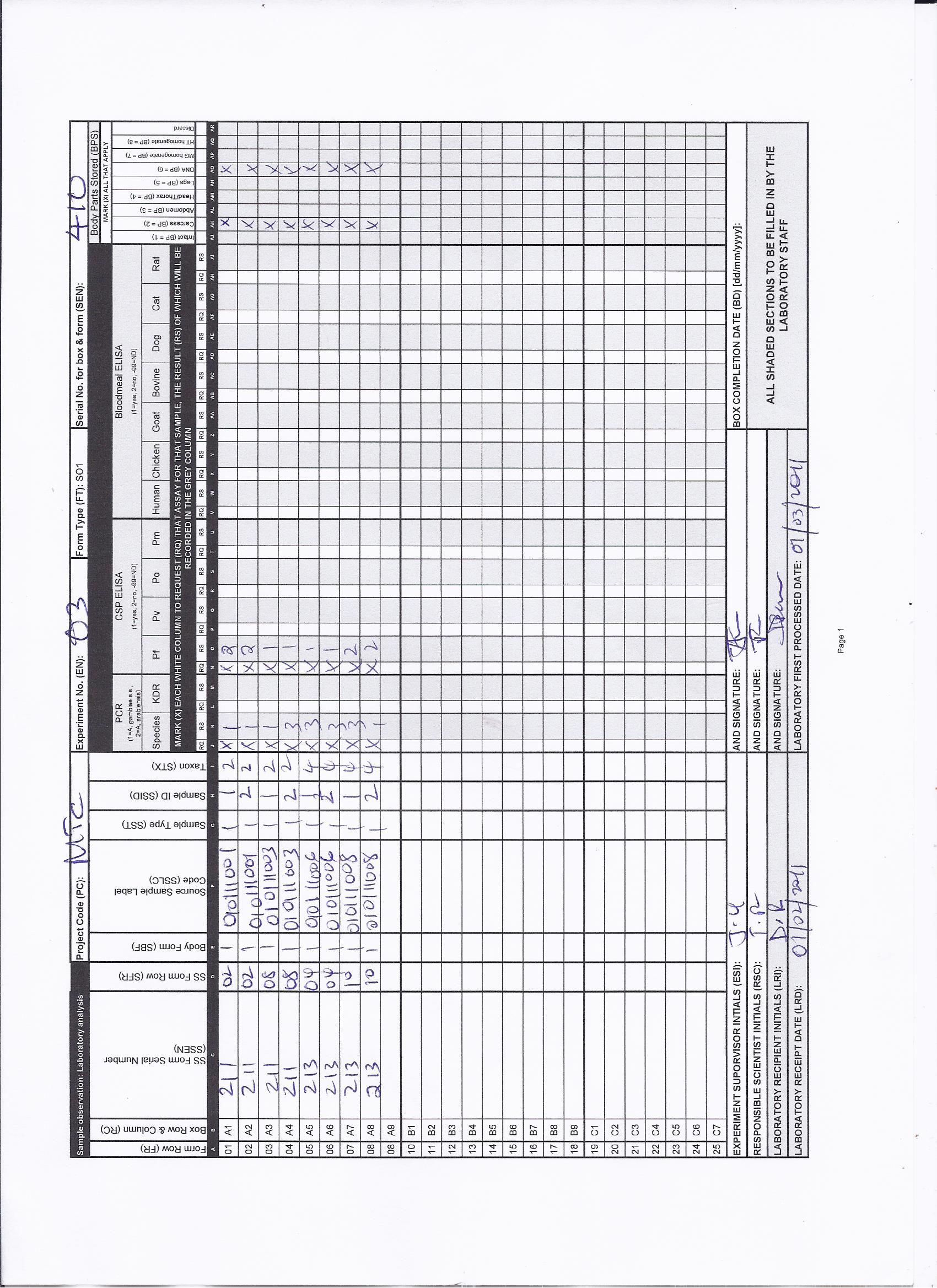
**

Figure S2 5: The SO1 form recording individual selected sample from SS1s forms with serial numbers 211 and 213, lab results for PRC and ELISA where the parts that need to be stored are marked on the far right side.

1. **Sample storage:** The DNA and carcasses of the mosquito samples were archived for long-term sample storage (marked on the far right side, Figure S2 5). The samples were placed into 81 cell storage boxes where storage of boxes in the laboratory was recorded using the *box record form* (ST).


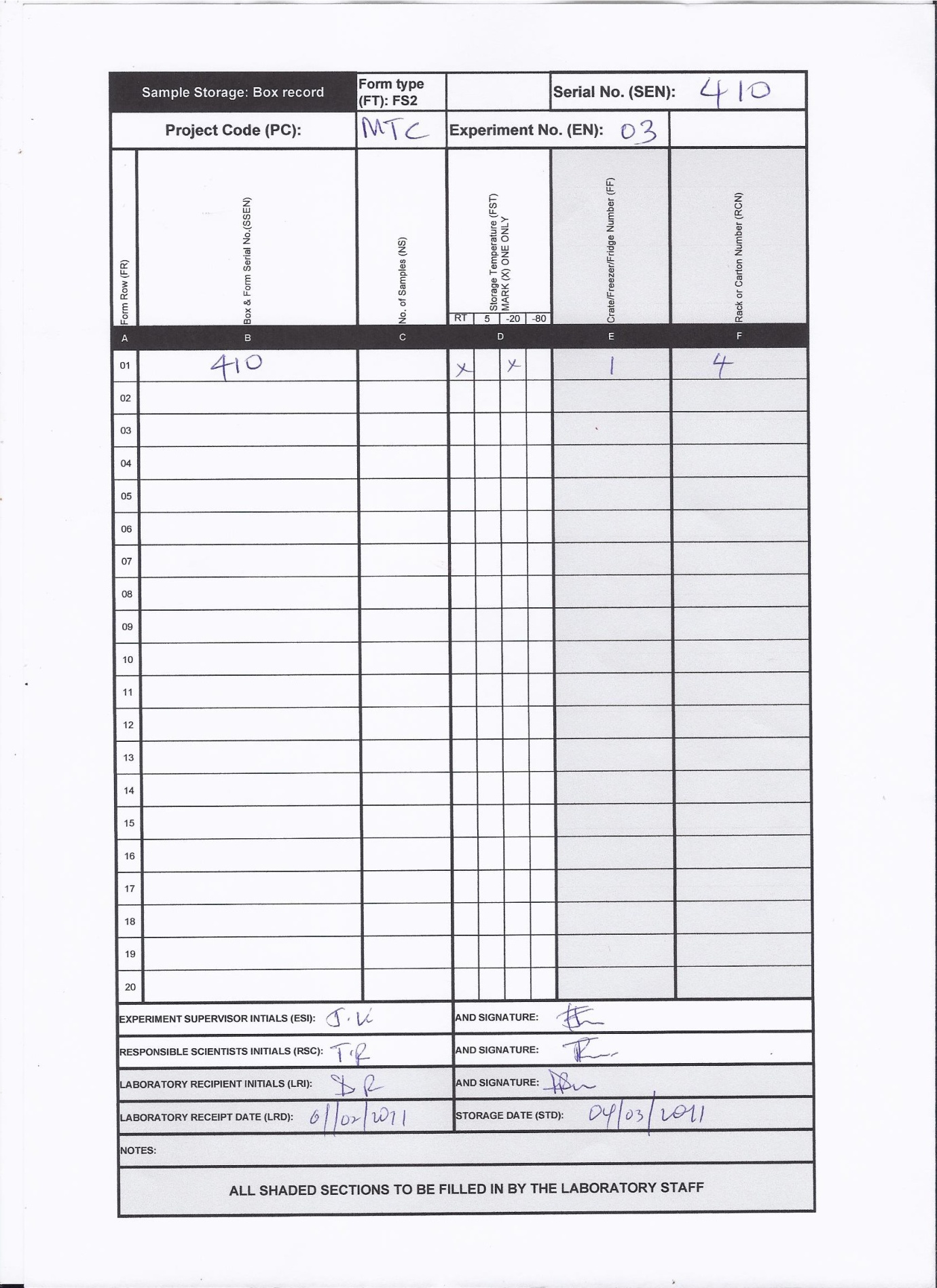


Figure S2 6: The ST2 form recording the storage information for samples such temperature, fridge and rack numbers (that way samples can be traced all the way to the storage facilities)

**Example 2: Survey of immature mosquitoes from natural field habitats**

The steps involved in the experiment were as follows:

1. ***Collect mosquitoes*:** One collection is the same as one trapping effort. Commonly, in the case of immature mosquitoes, one trapping effort may be up to ten 250ml dips. The details of the experimental design were recorded on the experimental design form (ED1). Data was recorded for the following collection attributes: *date of collection, enumeration area, cluster, compound or plot, household, method, indoor/outdoor, habitat type, start time,* and *finish time*.


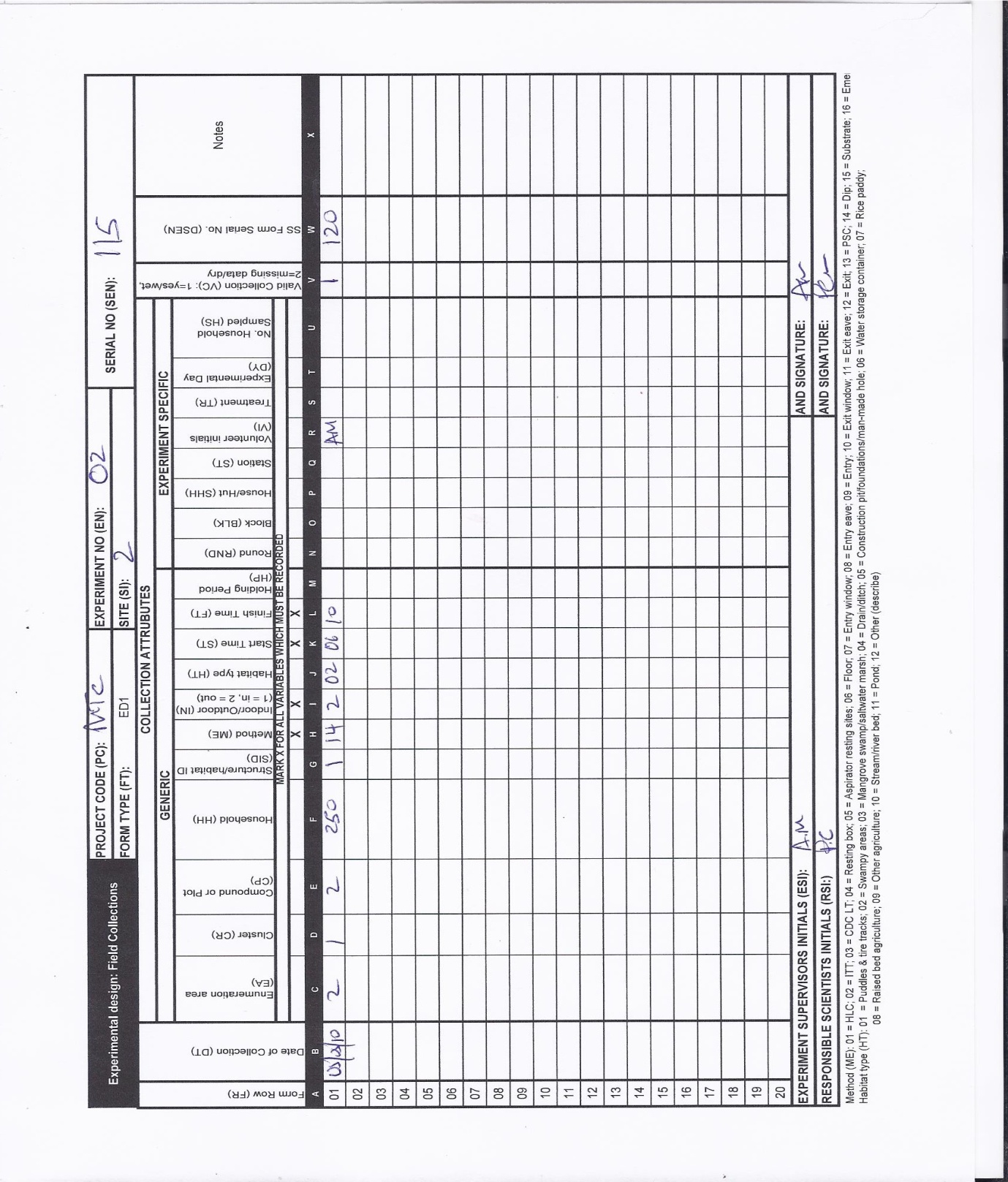


Figure 7: The ED1 form recording experimental information of immature collection where only the required attributes are filled-in with data.

1. ***Sort collection*:** Each collection was sorted into pre-defined subgroups of *taxon* and *body form*. These sample sorting attributes are categorical, as follows:
   1. Taxon: *Anopheles spp., Culex spp*.
   2. Body form: *instars 1 & 2, instars 3 & 4, pupae*

The number in each subgroup is counted and the data from each collection was recorded using one sample sorting form for immature field collection (SS2) (Figure S2 9).


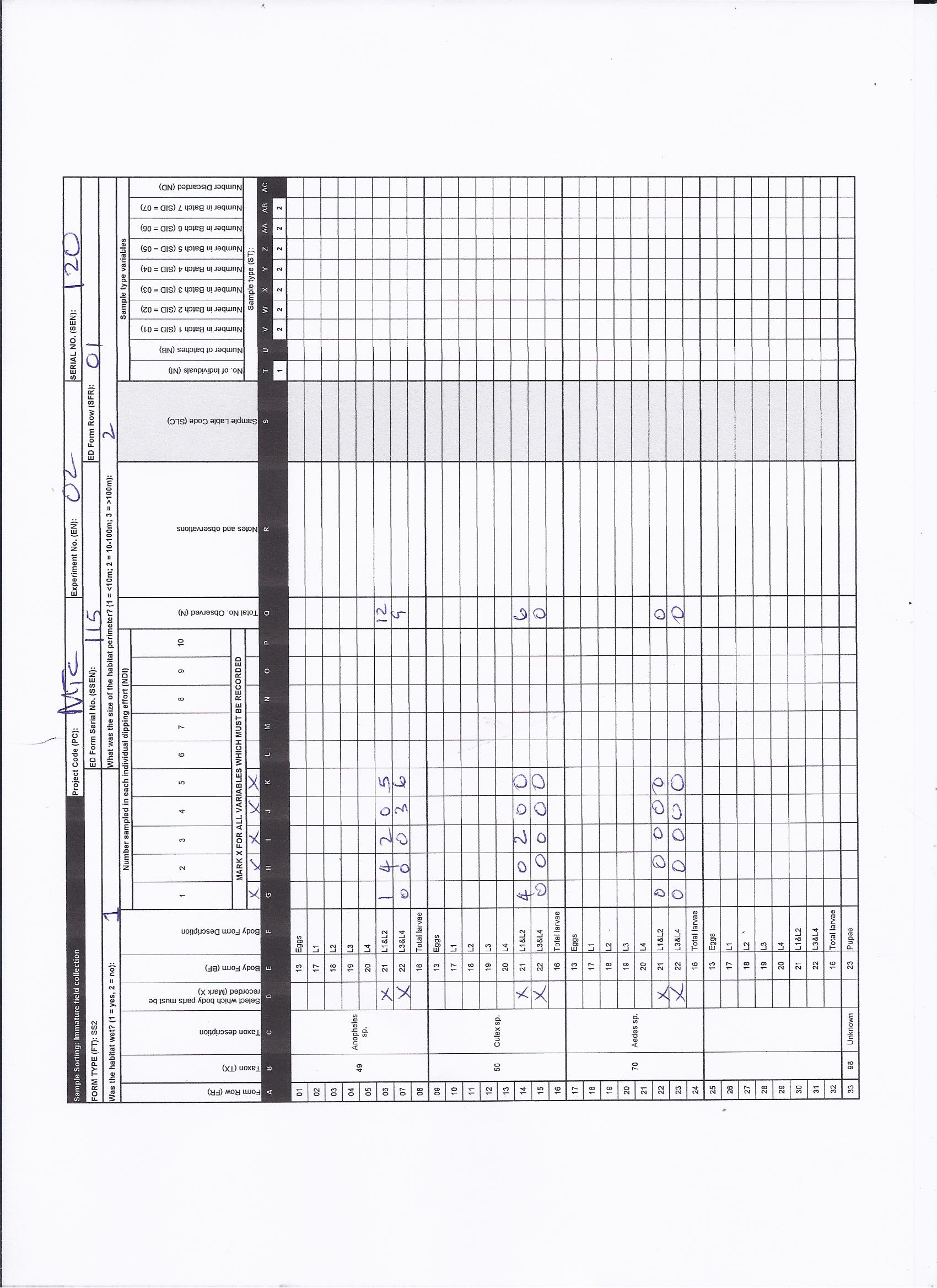


Figure S2 8: The SS2 form recording immature collection sorting information, indicating the number of observed for L1&L2 stages for different species.

1. ***Create samples*:** During the sorting process, samples of individual (n = 1) mosquitoes may be created, labelled and stored. The number of and labelling of samples is recorded on the corresponding sample sorting form (SS2) used in step 2.

**Example 3: Experimental hut assays of adult mosquito susceptibility to insecticide.**

The steps involved in the experiment were as follows:

1. **Collect mosquitoes**: one collection is the same as one trapping effort. Recorded using one line on the experimental design form (ED1). Data was recorded for the following collection attributes: *date, enumeration area (village), method, indoor/outdoor, start time, finish time, round, house/hut, volunteer initials, treatment* and *experimental day* (Figure 10).

Figure 9: ED1 form recording experiment design information for the required attributes, in this experiment, treatment = 10 represents untreated net and treatment = 11 is for treated nets.


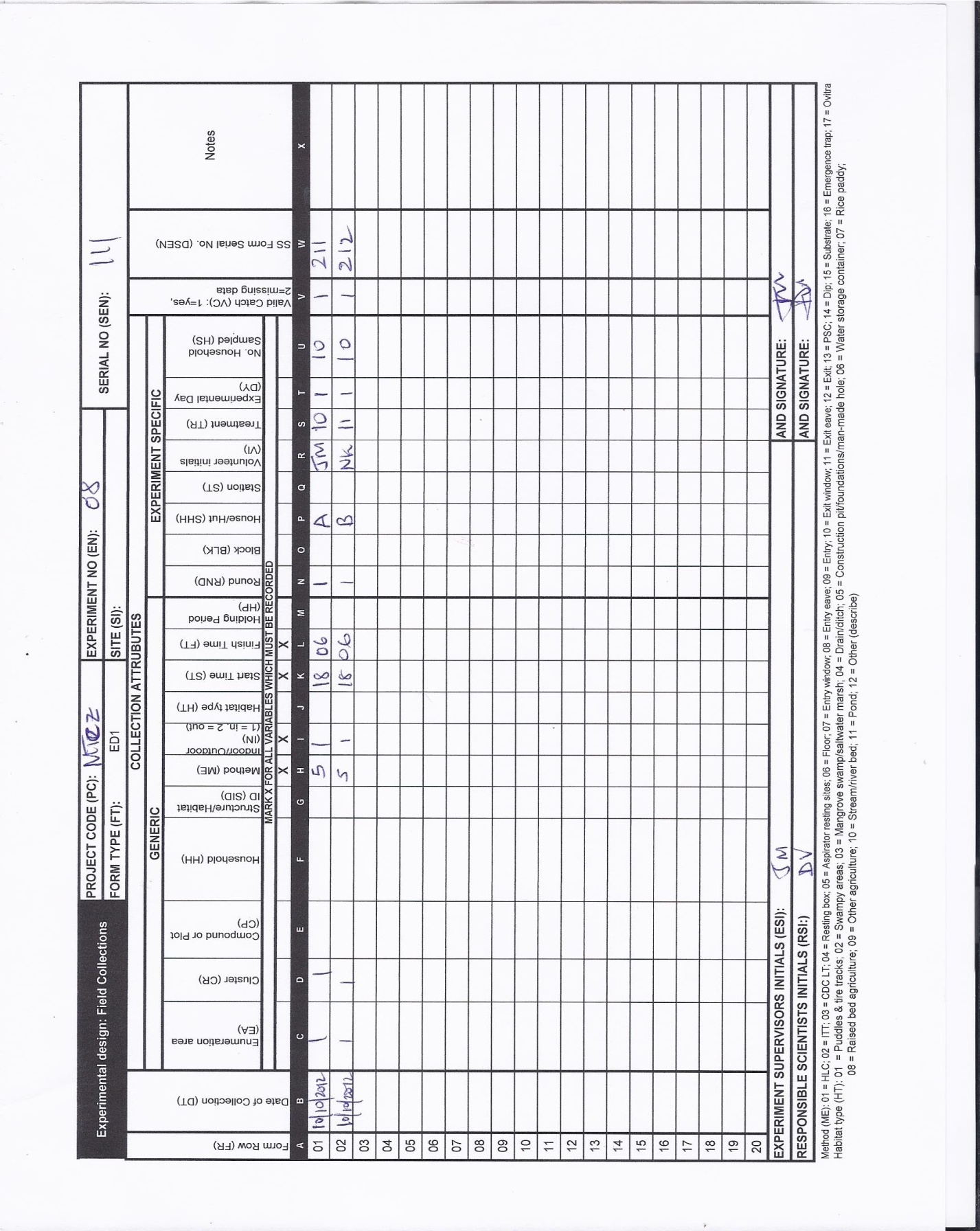


1. The mosquitoes were then kept for *24 h holding period* to observe delayed mortality.
2. **Sort samples:** Each collection was sorted into pre-defined subgroups of *dead, taxon, sex and abdominal status*. These sample sorting attributes are categorical, as follows:
   1. dead: dead, alive
   2. taxon: *A. gambiae s.l., A. funestus,* Culex spp., Aedes spp., Mansonia spp.
   3. sex and abdominal status: total male, unfed, part fed, fed gravid/semigravid, total female

Figure S2 10 SS3 : The SS3 form recording sorting information corresponding to treatment where untreated net was used


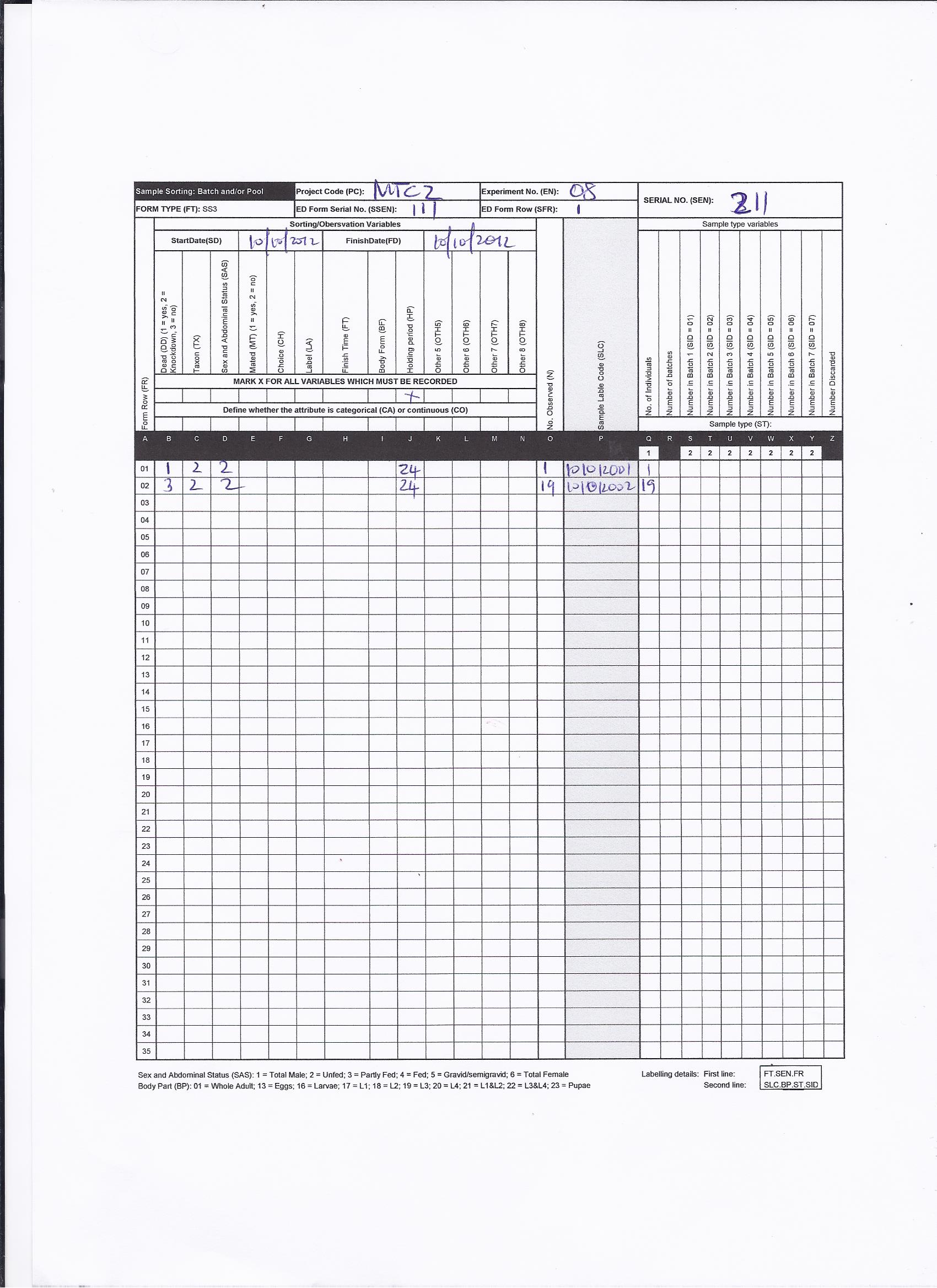


The number in each subgroup was counted, and the data from each collection was recorded using one sample sorting form for batch and/or pools (SS3) (Figure S2 11, Figure S2 12).

Figure S2 11: The SS3 form recording sorting information corresponding to treatment where treated net was used


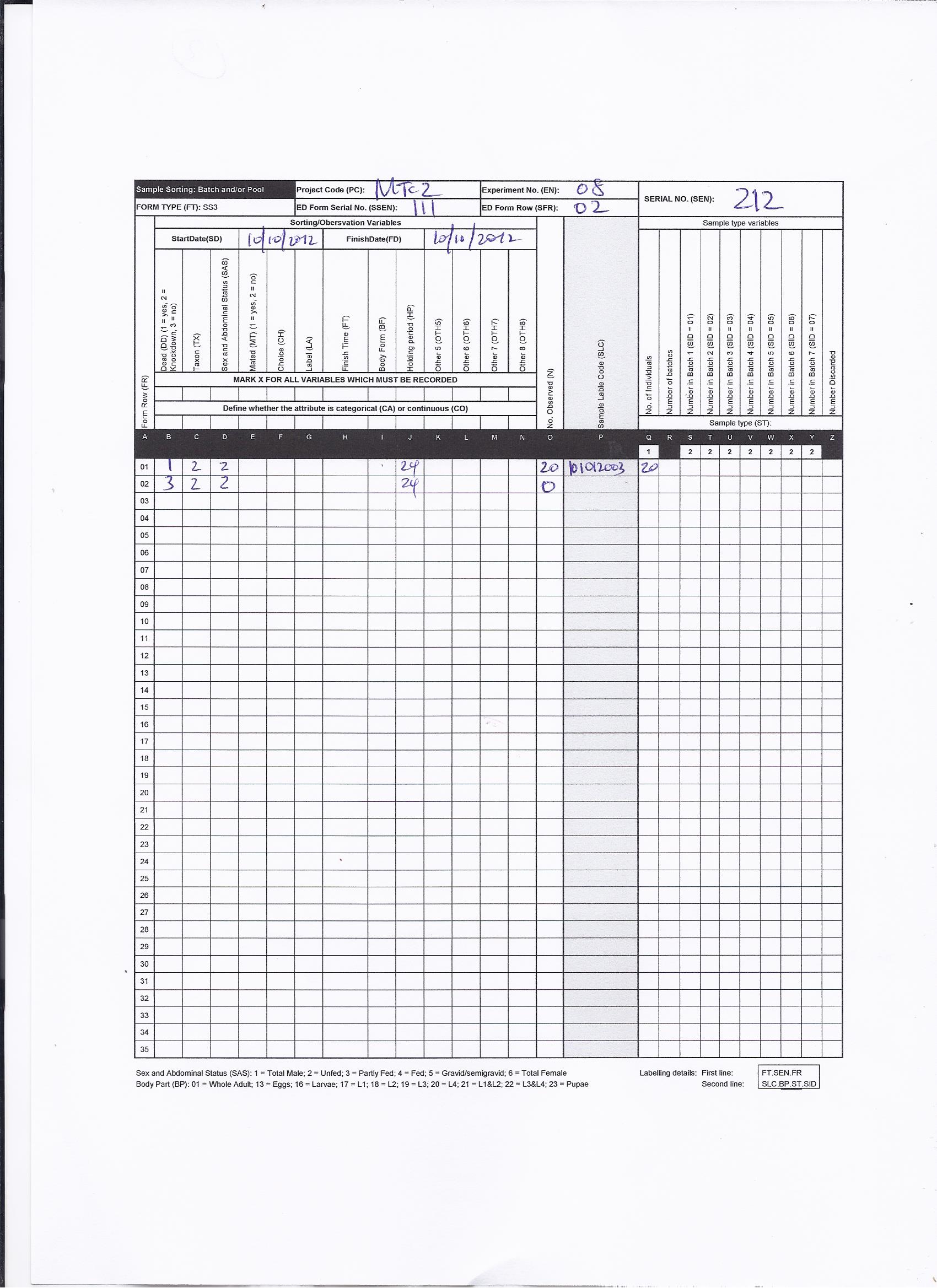


1. **Create samples:** during the sorting process, samples of individual mosquitoes are created, labelled and stored. The number of and labelling of samples is recorded on the corresponding sample sorting form (SS3) used in step 3.

The infection status and sibling species identity of samples was observed using ELISA and PCR and recorded on SO1 forms using the same procedures as described in example 1. Also, the carcasses of the mosquito samples are archived for long-term sample storage using the ST form.

**Example 4: Insecticide susceptibility bioassay under laboratory conditions**

The steps involved in the experiment were as follows:

1. **Experimental design:** Batches of live mosquitoes are collected from a specified colony in the insectary. The details of the experimental design were recorded on the experimental design form (ED2). Data was recorded for the following collection attributes: *colony code, sex and abdominal status, age, number of mosquitoes, start date, treatment,* and *replicate*.

Figure S2 12: The ED2 form recording experimental design information from insectary, as an example treatment = 50 represent control where treatment = 51 represent concentration of fungal conidia, each with three replicates.


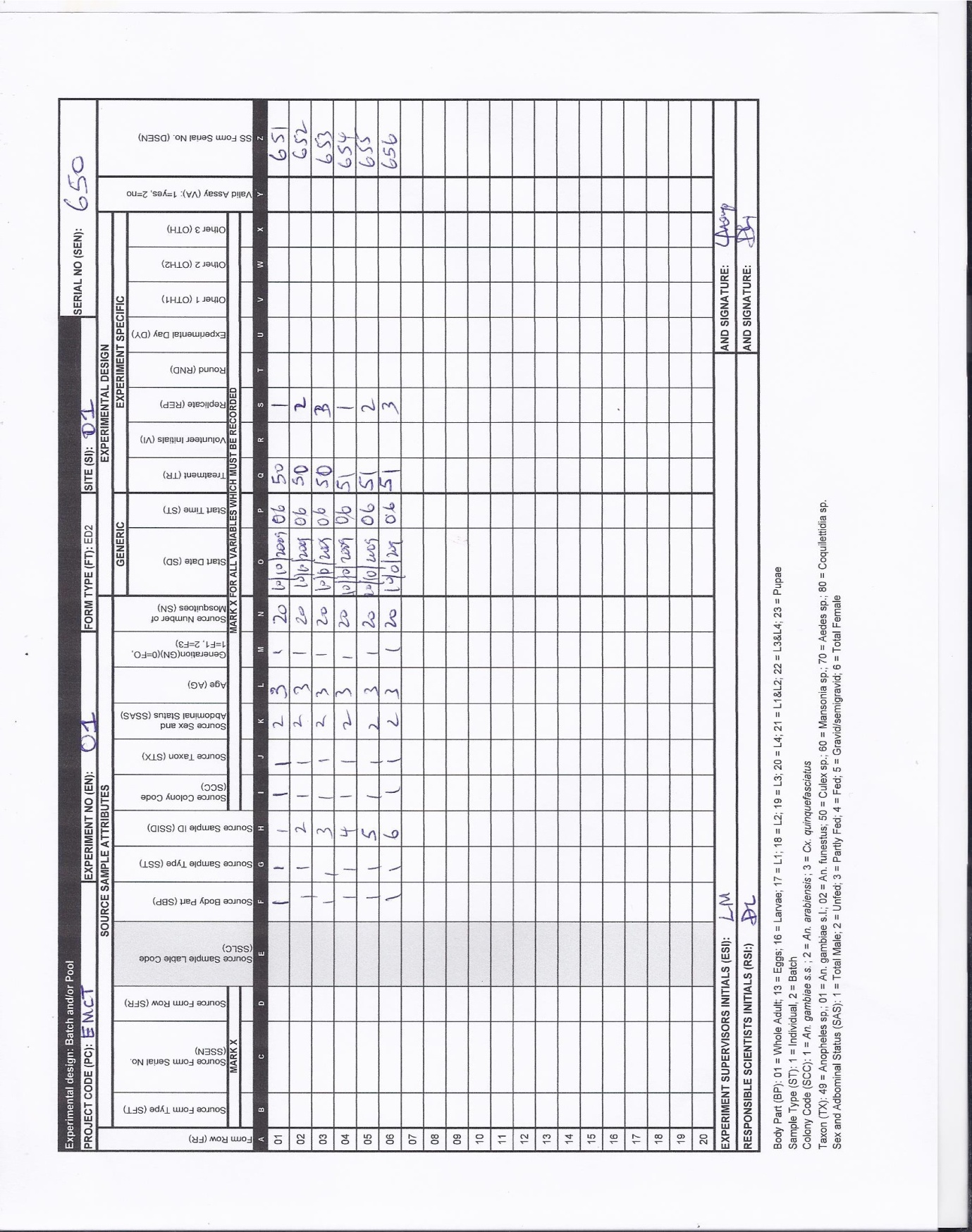


1. **Sample sort:** each collection was sorted into pre-defined subgroups of dead and finish date. These sample sorting attributes are categorical, as follows:
   1. dead: dead, alive
   2. finish date: the date for which the experimental sorting was completed and used to define each 24 h holding period

Figure S2 13: The SS3 form recording sample sorting corresponding to the first row of ED2, control first replicate.


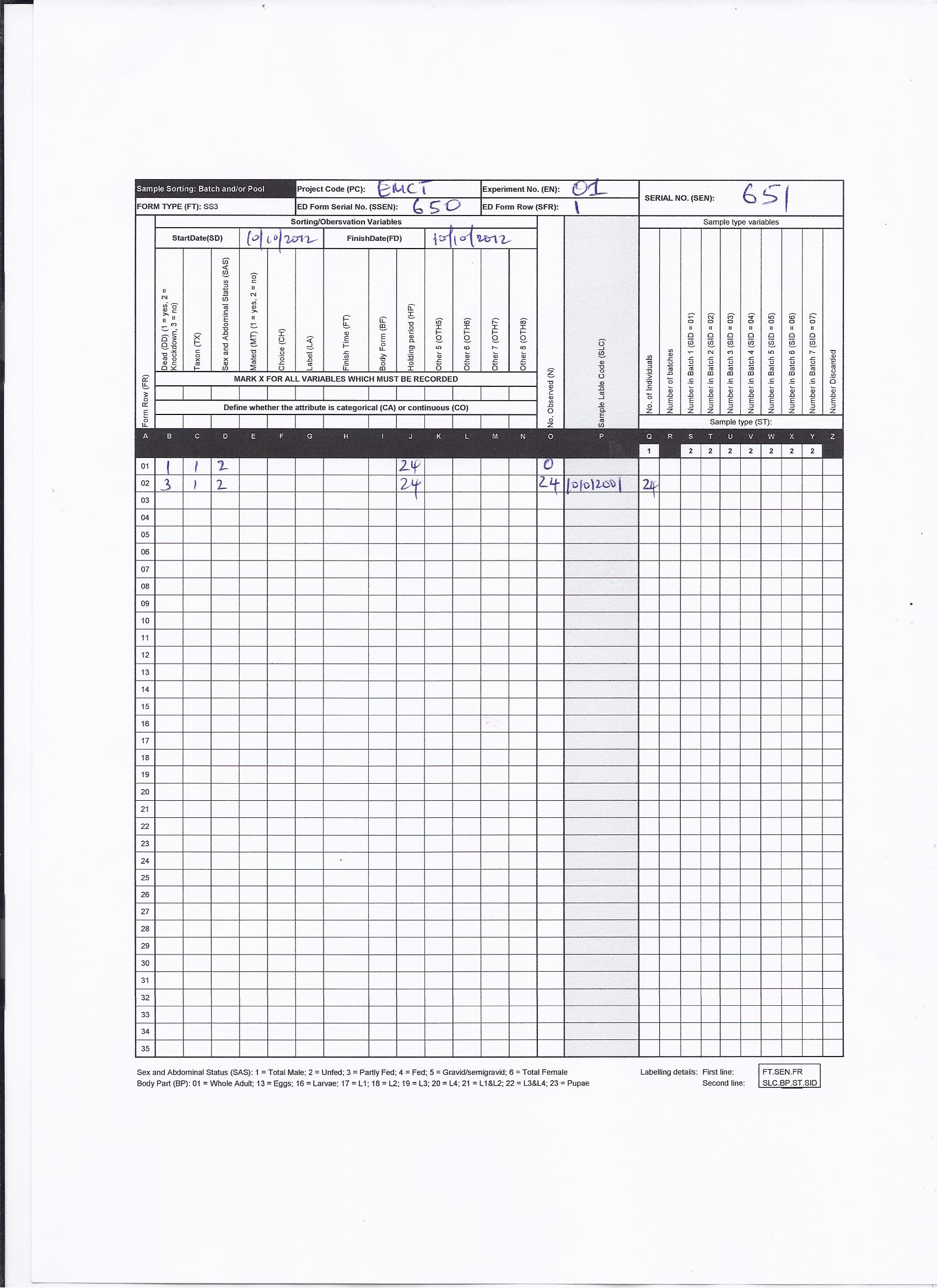


The number in each subgroup was then observed by counting the number of individuals, and the data from each collection was recorded using one sample sorting form for batch and/or pools (SS3), as example one for one control replicate Figure S2 14 and another one for one treatment replicate Figure S2 15.

~~
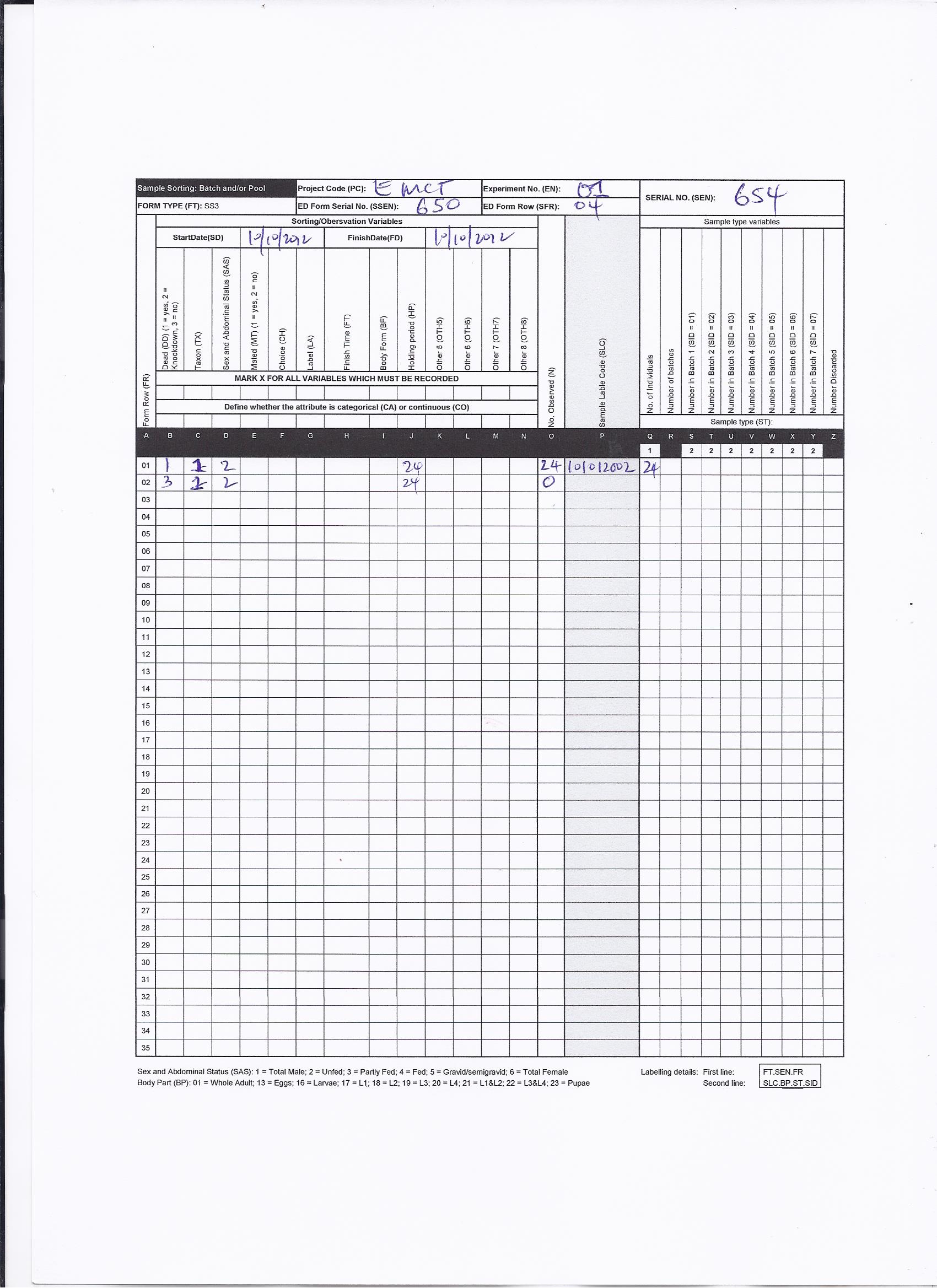
~~

Figure S2 14: The SS3 form recording sample sorting corresponding to the fourth row of ED2, treatment first replicate.

**Example 5: An experiment to determine the residual efficacy of ACTELLIC^®^ 300 CS, Pirimiphos-methyl in two malaria endemic areas in Zambia.**

Indoor Residual Spraying remains the cornerstone of malaria prevention and control in Zambia. To ensure that this transmission-blocking tool remains effective, and its choice is evidence based, monitoring the effectiveness of IRS products is essential. The WHO cone bioassays were used to assess the residual efficacy of pirimiphos methyl (ACTELLIC^®^ 300 CS), an organophosphate, at 1g/m^2^ for indoor residual spraying on cement and mud rendered walls inside human houses using non-blood fed *Anopheles gambiae s.s* in Zambia.

The steps involved in the experiment were as follows:

1. **Experimental design:** Batches of live mosquitoes were collected from a colony of *Anopheles gambiae s.s* maintained at the National Malaria Control Centre insectary. The details of the experimental design were recorded on the experimental design form (ED2). Data was recorded for the following collection attributes: *colony code, taxon, sex and abdominal status, age, number of mosquitoes, start date, start time, treatment, replicate, and destination serial number.*

Figure S2 15: The ED2 form recording experimental design information from insectary, in this experiment, treatment = 10 represent control environment where treatment = 12 represent pirimiphos methyl.


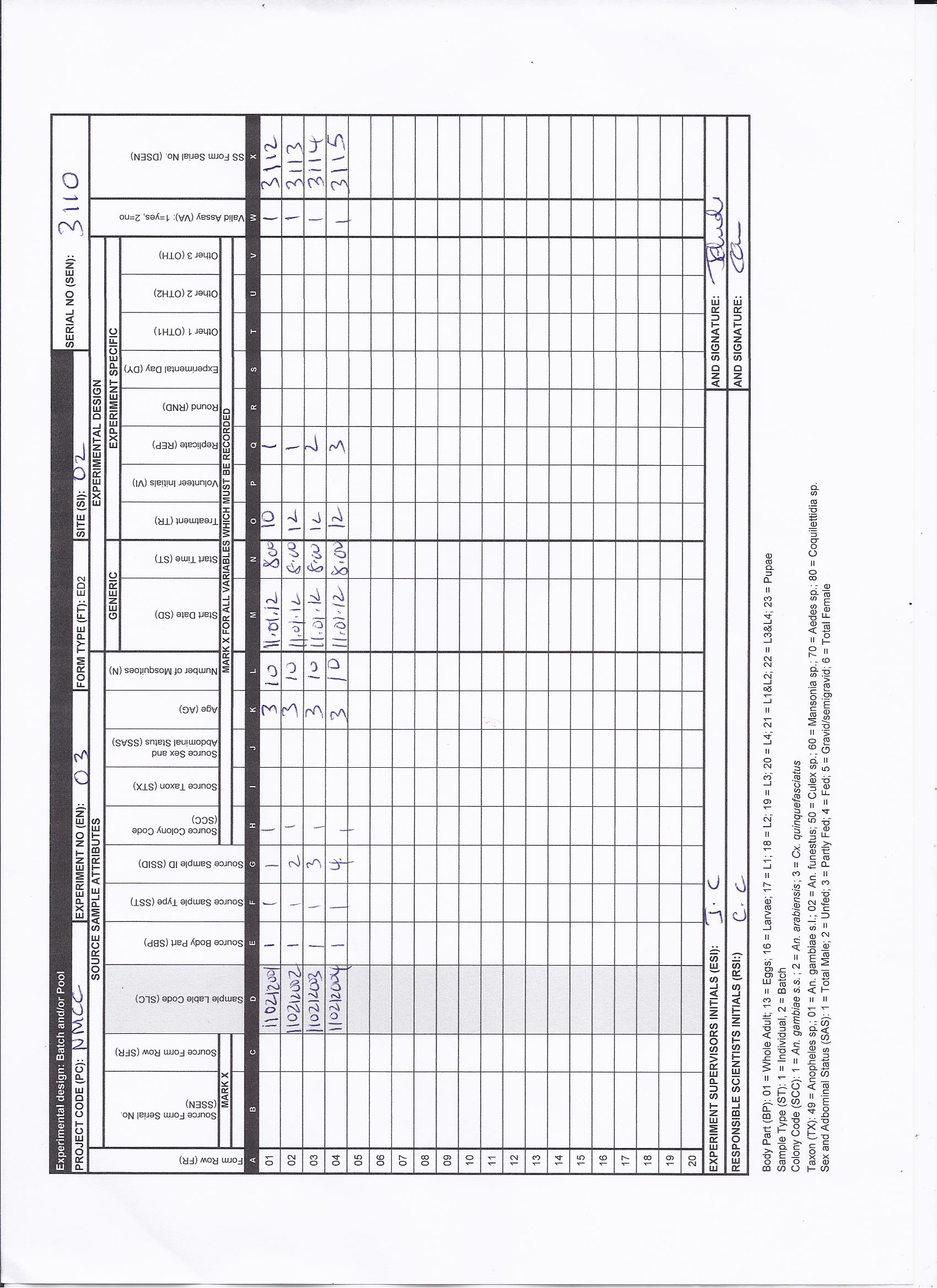


1. **Sample sort:** Each bioassay test collection was sorted into pre-defined subgroups of dead and finish date. These sample sorting attributes are categorical, as follows:
   1. dead: dead, alive
   2. finish date: the date for which the experimental sorting was completed and used to define each 24 h holding period

Figure S2 16: The SS3 form recording sample sorting corresponding to the first row of ED2, control first replicate.


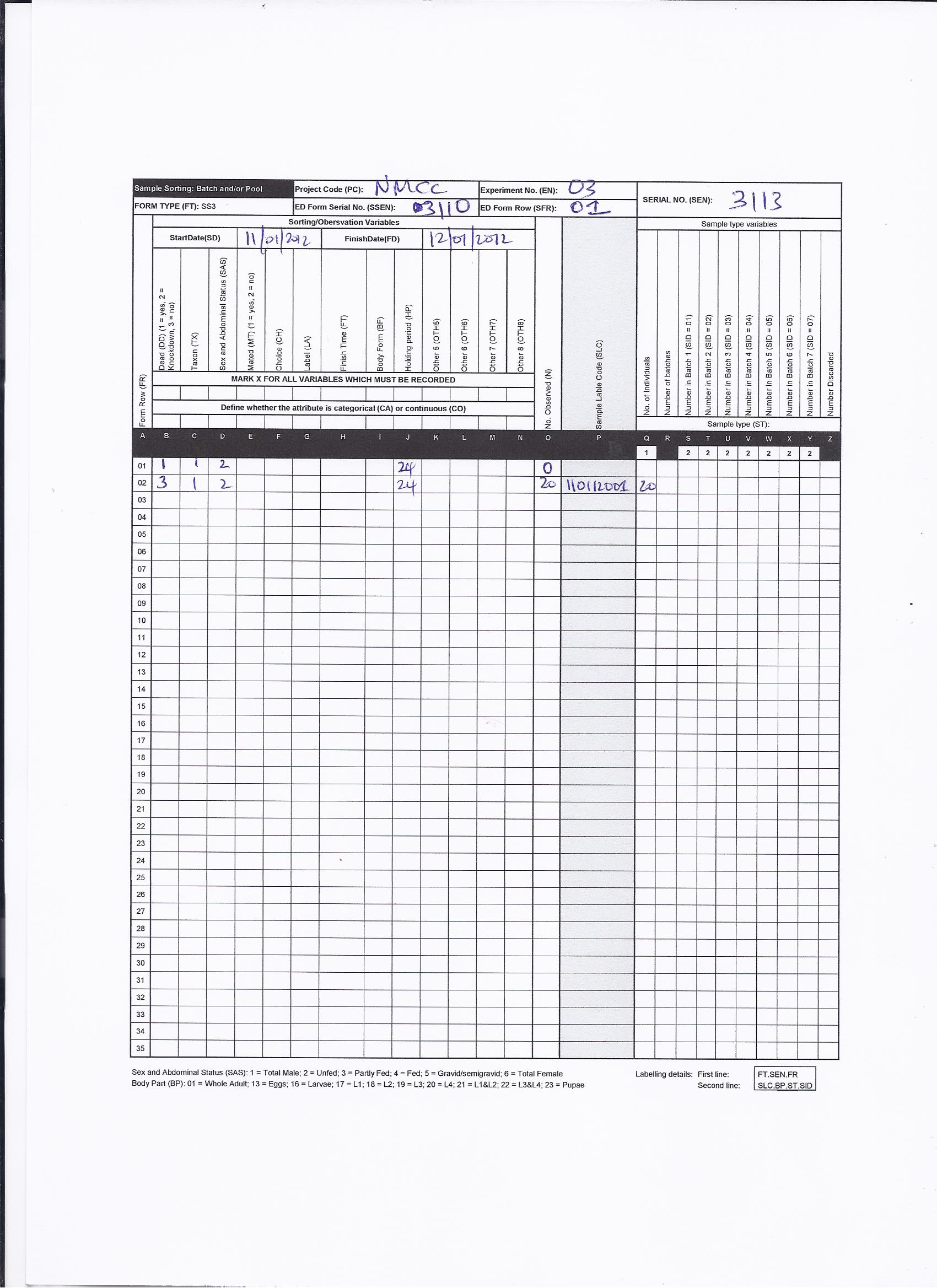


1. **Sample observation:** The number in each bioassay was then observed by counting the number of individual mosquitoes exposed to the insecticide and the control after 60 minutes of exposure period according to the WHO standard protocol of 2013. The mosquitoes were placed in holding paper cups with access to 10% sugar solution and mortality rates were scored after 24 hour (1440 minutes). The data was then recorded for each assay using one sample sorting form for batch and/or pools (SS3), (first control shown as an example in Figure S2 16) with following collection attributes: *form row , dead ( 1=yes, 2=knockdown, 3=no), taxon, holding period , number observed , sample label code, and number of individuals*.

**Example 6: An experiment to monitor insecticide resistance and malaria vector infection status**

This was a longitudinal study conducted to measure the effect of insecticide resistance on malaria vector infectivity rates in Luangwa, Zambia. Adult indoor resting mosquitoes were collected using a CDC back pack aspirator from ten houses for 4 days each from 4 am to 7 am. Mosquitoes were put in one cage and were carried to the Laboratory to obtain first filial generation (F1) needed for insecticide susceptibility tests while field mosquitoes (parents, F0s) that laid eggs were screened for sporozoite infectivity using ELISA method at the molecular laboratory.

The steps involved in the experiment were as follows:

1. **Mosquitoes Collection**: Adult mosquitoes are collected and are placed into one collection cage and recorded using one line on the experimental design form (ED1) Figure S2 17.

. **
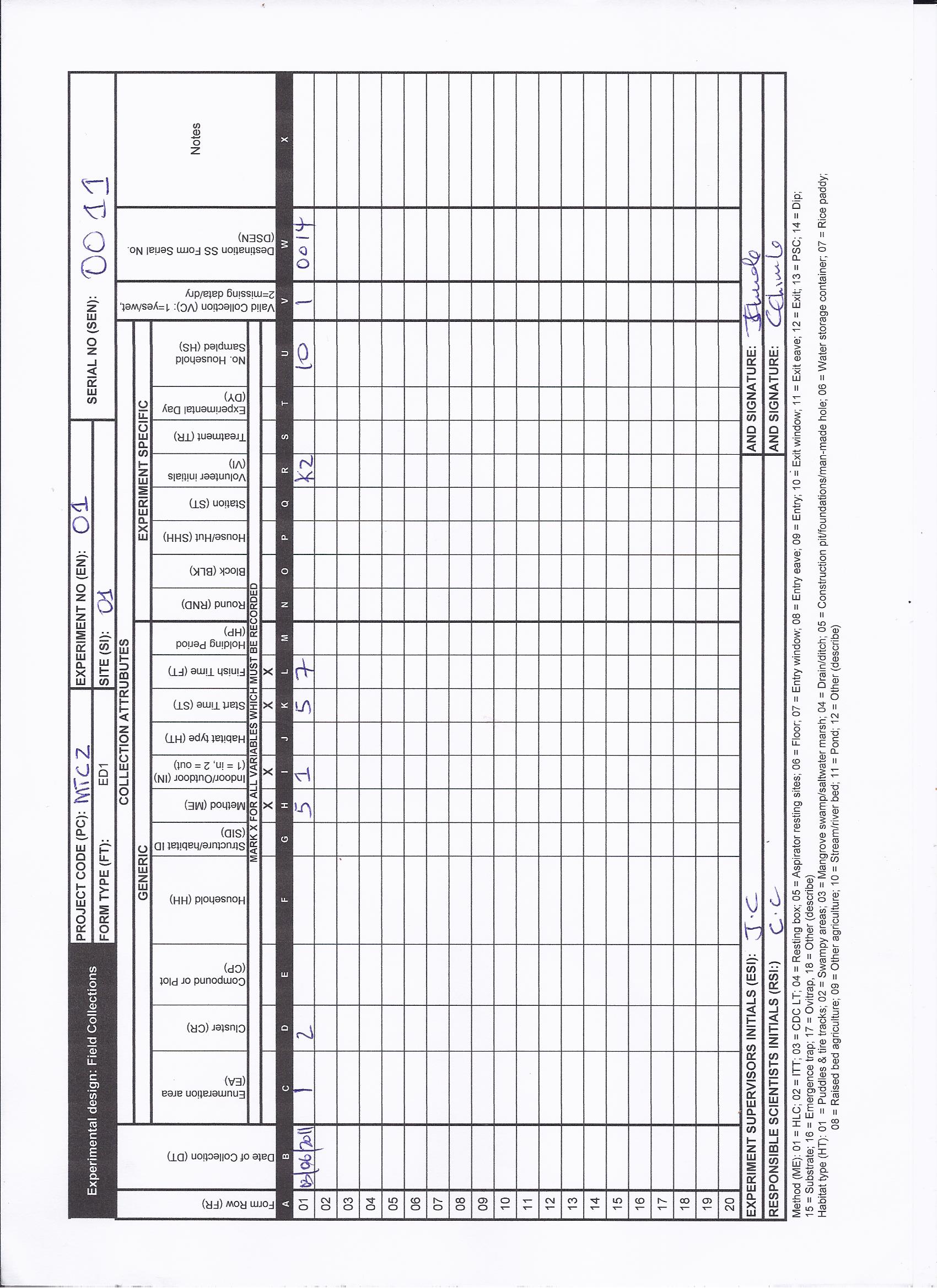
**

Figure S2 17: The ED1 form recording experiment for the resistance experiment where 10 houses were sampled to obtain the first generation of mosquitoes.

Data was recorded for the following collection attributes: d*ate of collection, enumeration area (village), cluster, method, indoor/outdoor, start time, finish time, volunteer initials, number of households sampled, valid collection (*to indicate if the collection was valid or not), *and destination SS form serial number (to indicate the serial number of the sorting form).*

1. **Sort collection: Sample observation:** Each collection of adult mosquitoes was sorted into pre-defined subgroups of *taxon, sex* and *abdominal status*. Blood fed and partially gravid mosquitoes were kept for 2 to 3 days to become fully gravid. Each gravid mosquito was transferred into an eppendorff tube containing a moist filter paper to allow forced egg oviposition. The F0 generation mosquitoes were sorted morphologically and recorded according to the *taxon, sex & abdominal status, number of mosquitoes caught, number*

*of individual samples* stored using the sample sorting form (SS1) (Figure S2 18).


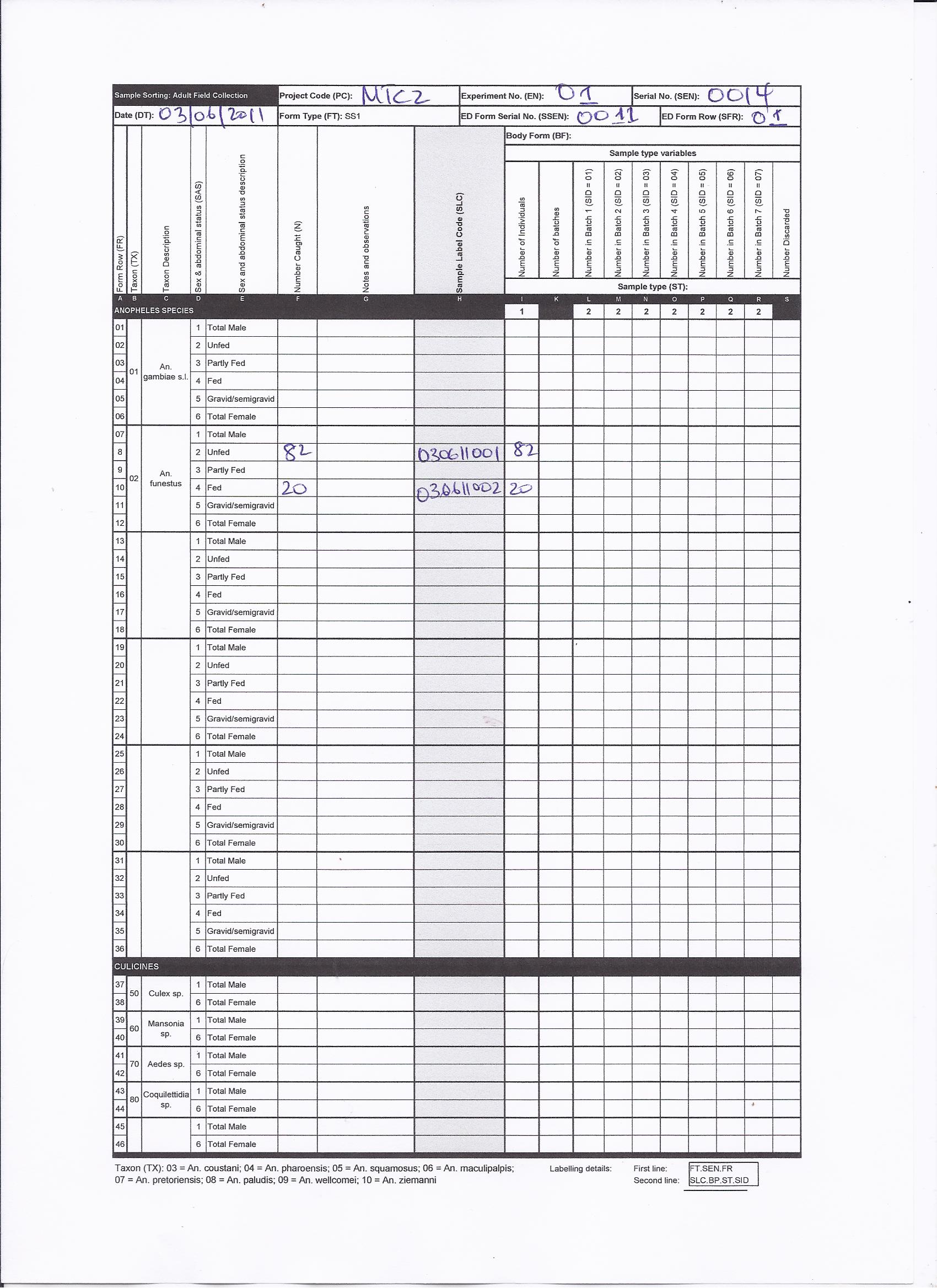


Figure S2 18: The SS1 form recording sample sorting information corresponding to first ED1 row of mosquitoes sampled from 10 houses.

1. **Sample observation:**  The adult F1 generation progeny of mosquitoes were reared in the laboratory at Zambia’s National Malaria Control Centre. The mosquitoes were separated into male and female, counted and recorded in the experiment with the following attributes: *form row, source form serial number, source form row, sample label code, body form, sample type, sample ID, taxon, sex and abdominal status, age, generation, number of mosquitoes, start date, start time, treatment, replicates valid collection, and destination serial number* using experimental design: batch and/or pool form (ED2).


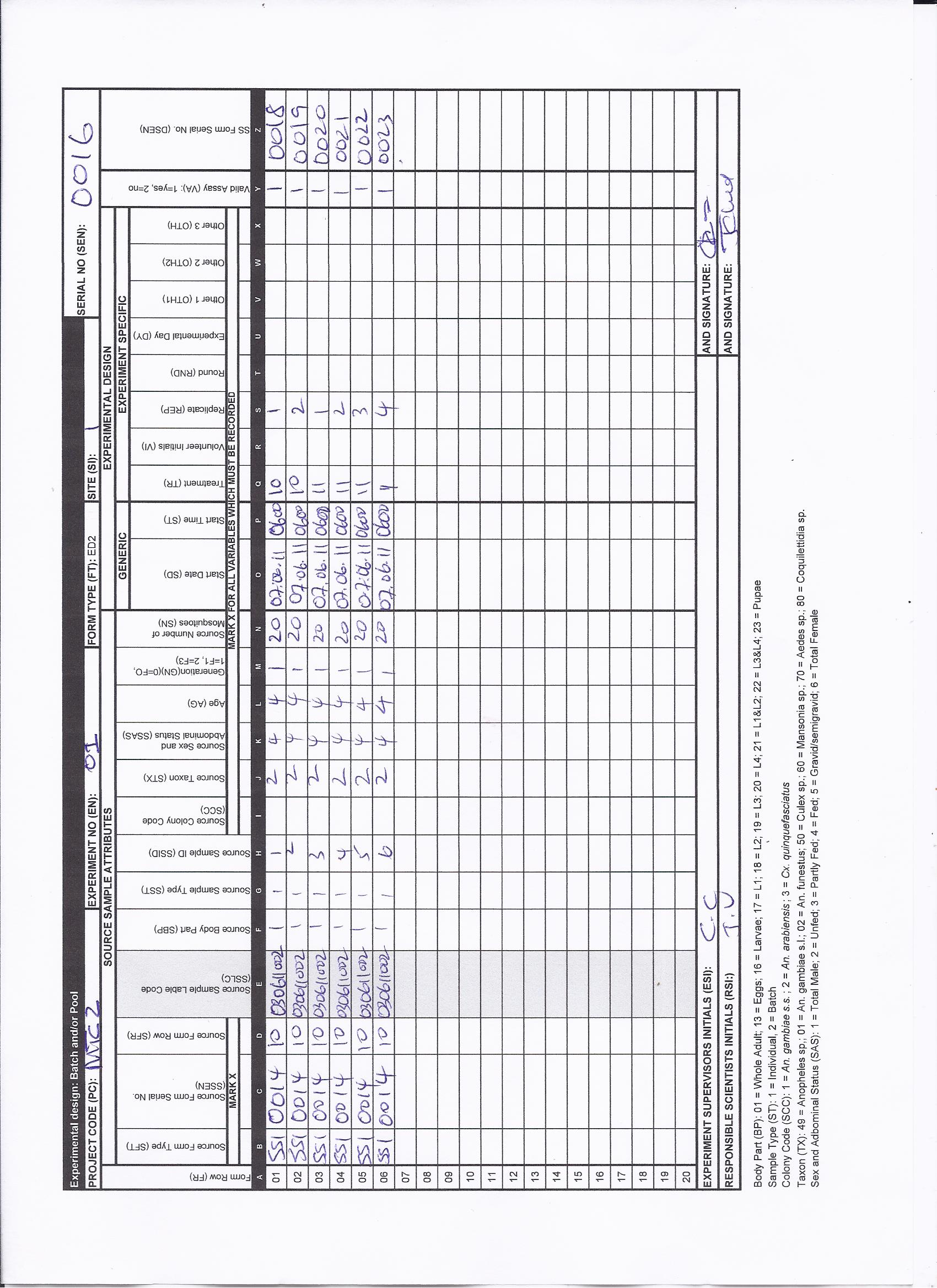


Figure S2 19: The ED2 form recording experimental design for generating F1 generation of mosquitoes using the parents from the field Figure S2 17 sorted using SS1 form Figure S2 18. to obtain mosquitoes that can be used for insecticide resistance experiment assay.

1. **Sample observations**: The newly emerged 2-5 days old F1 generation adult female mosquitoes were exposed to a pyrethroid (deltamethrin) and two controls for a period of 60 minutes. During the exposure period, knockdown time was recorded every 10 minutes and mortality rate was scored after 1440 minutes (24 hours) per WHO recommendation on insecticide resistance assay (WHO 2013). The data on exposed mosquitoes was recorded following the collection attributes: *dead (1 = yes, 2 = knockdown, 3 = no), taxon, holding period, number observed, sample label code,* and *number of individuals* using the sample sorting: batch and /pool form (SS3) (Figure S2 21).

Figure S2 20: The SS3 recording sample sorting information for the first row of ED2 in Figure S2 19. Another SS3 form is required for recording sample sorting for the rest of the rows.


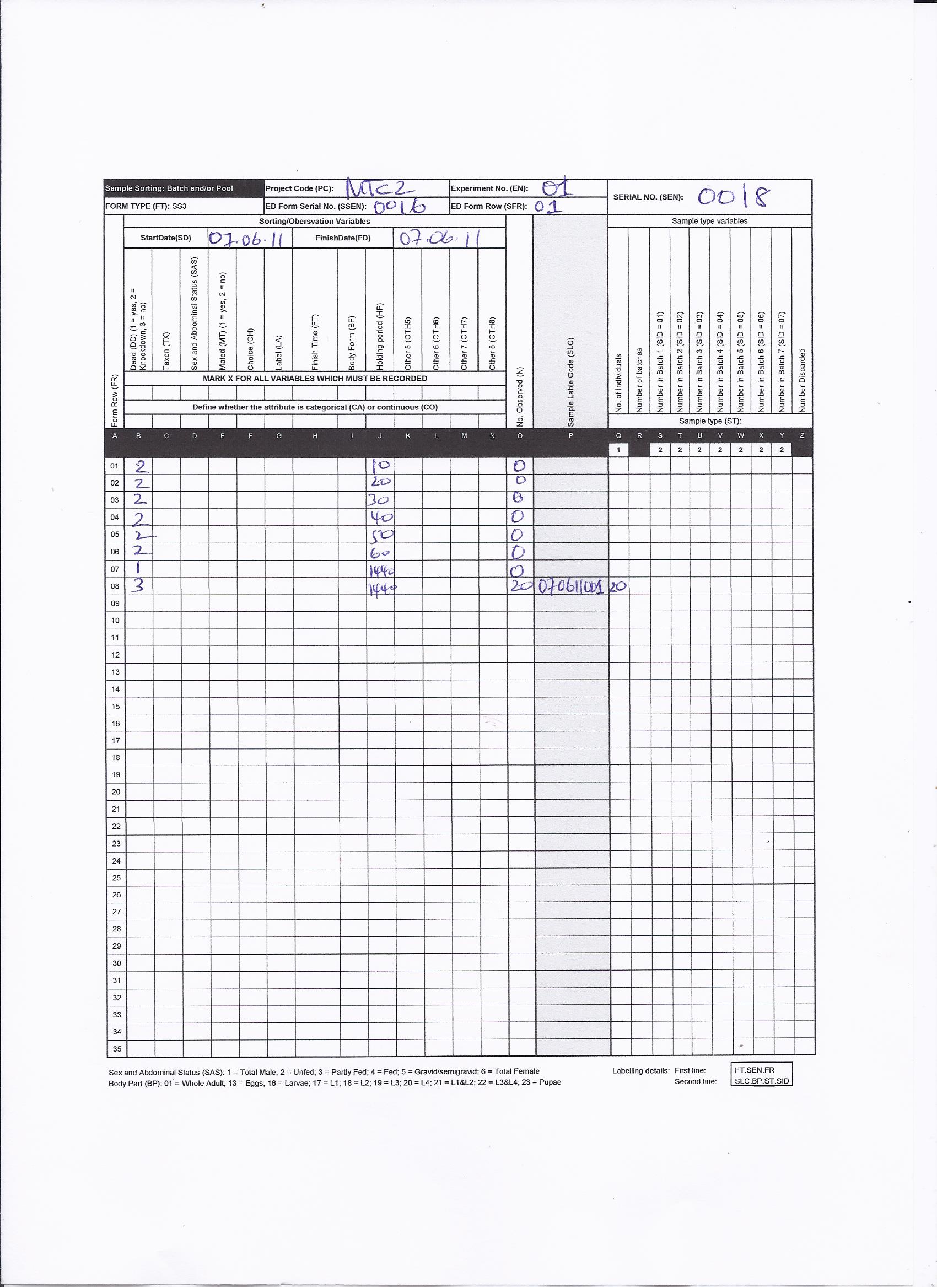


1. **Sample observation:** The prevalence of *sporozoites* in each individual mosquito sample that laid eggs for resistance measurements was measured using ELISA method while PCR was used to identify vector *sibling species* collected from the field and recorded on a sample observation form for laboratory assays (SO1) (Figure S2 22).

**
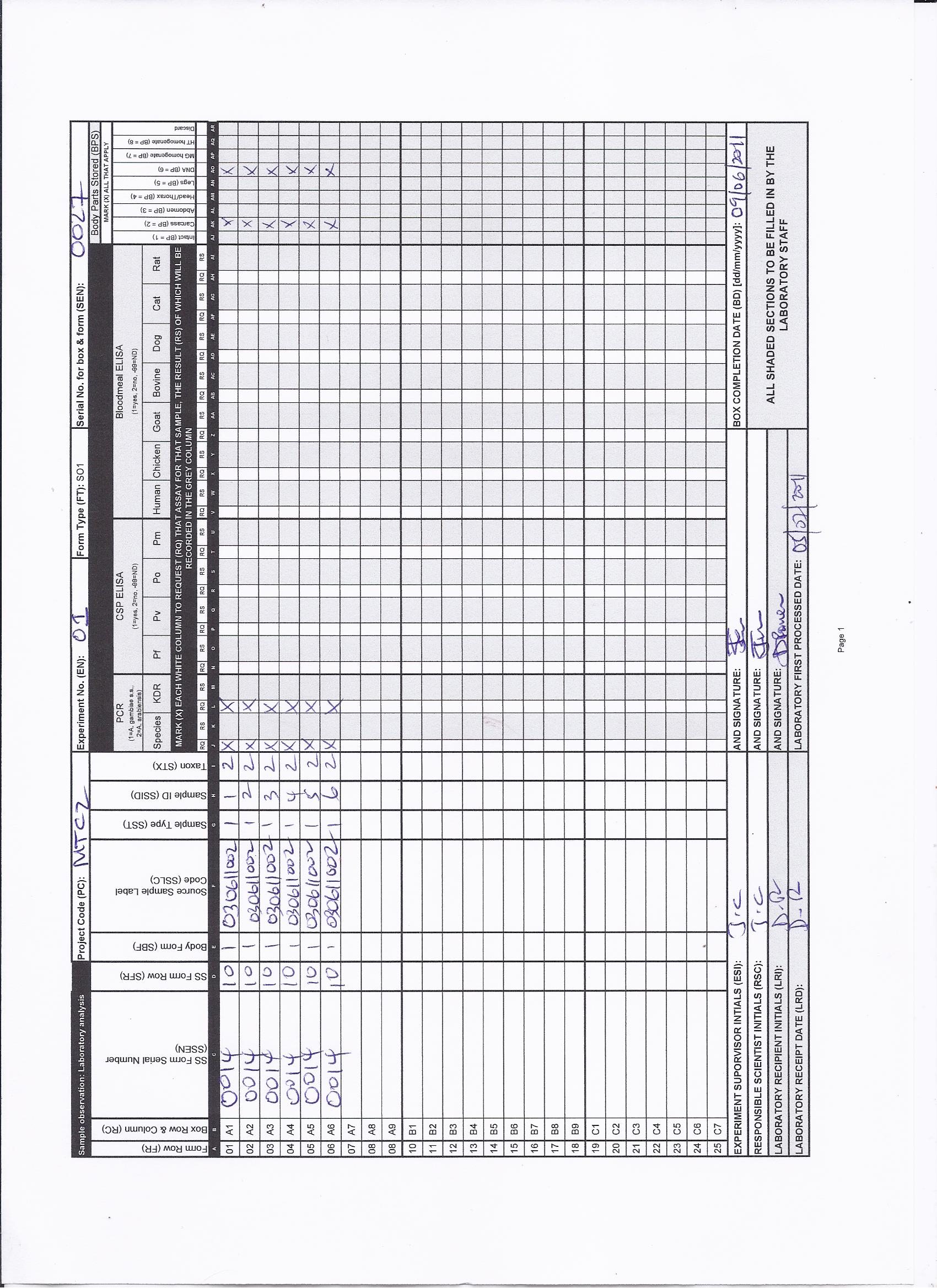
**

Figure S2 21: The SO1 recording scientific observation for F0 generation as sampled in SS1, only few samples are listed as an example. The parts marked on the far right side will be stored.

1. **Sample storage:** The DNA extracted from the heads and thoraxes of each individual mosquito sample were placed into 81 cell storage boxes for storage. The samples storage information was recorded using the storage of boxes in the laboratory using the *box record form* (ST1).

**
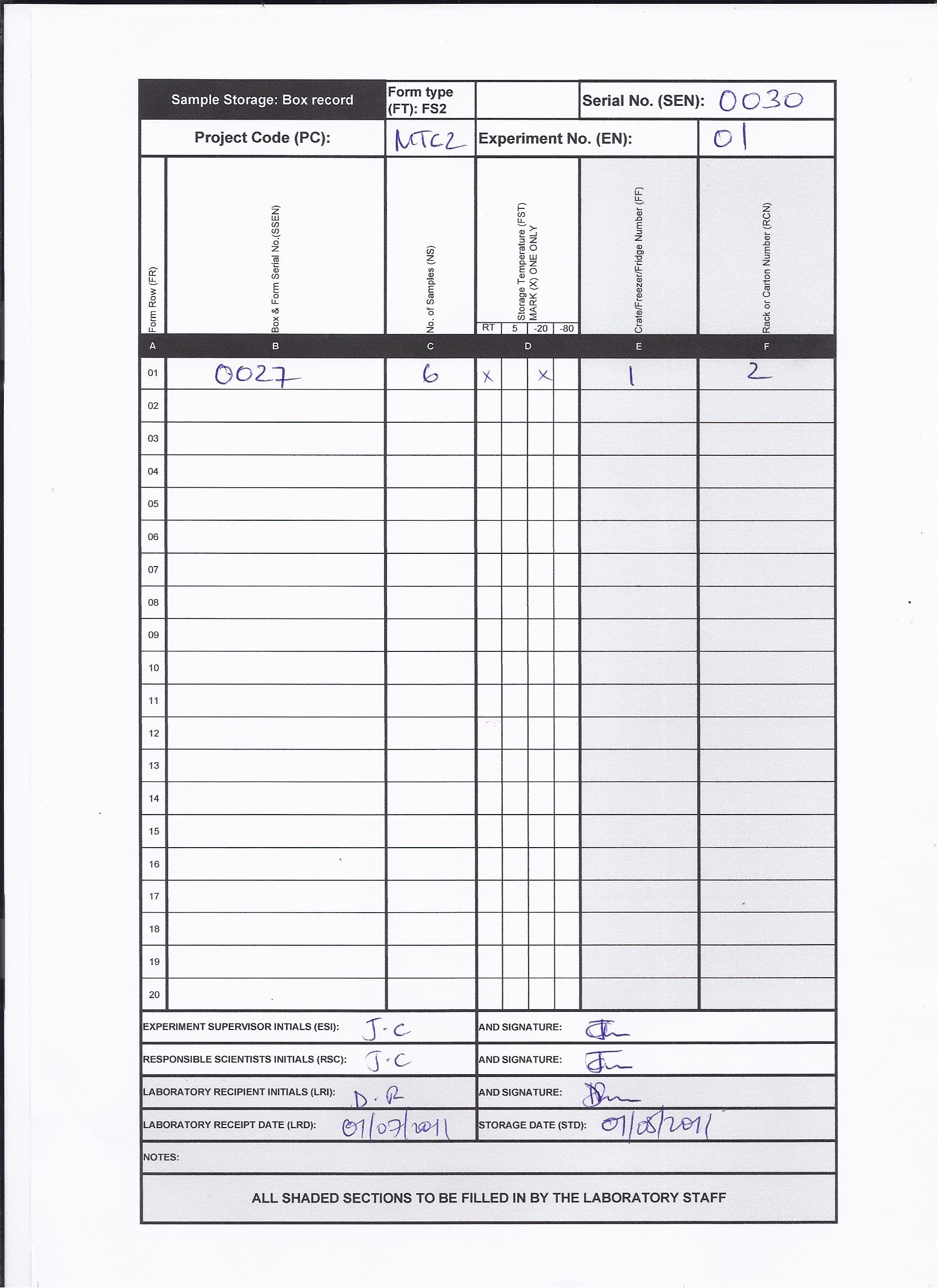
**

Figure S2 22: The ST2 form recording storage information for F0 generation.

Example 7: An experiment to analyze the host attraction of annopheline mosquitoes in Zambia.

The ordinary tent traps with host (human, cow, and goat) baits were used to analyze the attraction of annopheline mosquitoes in Luangwa, Zambia. These tent traps consist of a light frame covered by mosquito netting or thin durable cloth with a zip-able door to allow mosquitoes to enter. A human placed in the tent was protected with untreated mosquito net to avoid any exposure to mosquito bites and was given prophylaxis before the start of the experiment. A 3x3 cross over design was used to test whether there is a difference in the number and type of annophelines attracted to human, cow, and goat. The baits were rotated through three locations.

The collection of mosquitoes is done from 19hrs to 7hrs where the rotation is repeated for a total of 9 nights. The walls inside the tents are inspected hourly for resting mosquitoes collected with an aspirator. A tent used for a particular host was designated for use only with that host to avoid any lingering attractiveness or repellent effects that may confound the data.

Figure S2 23: The ED1 form recording experimental design for human as a bait from 19:00 to 6:00 for the first round. Another ED1 forms can be used for other baits and other rounds.

**
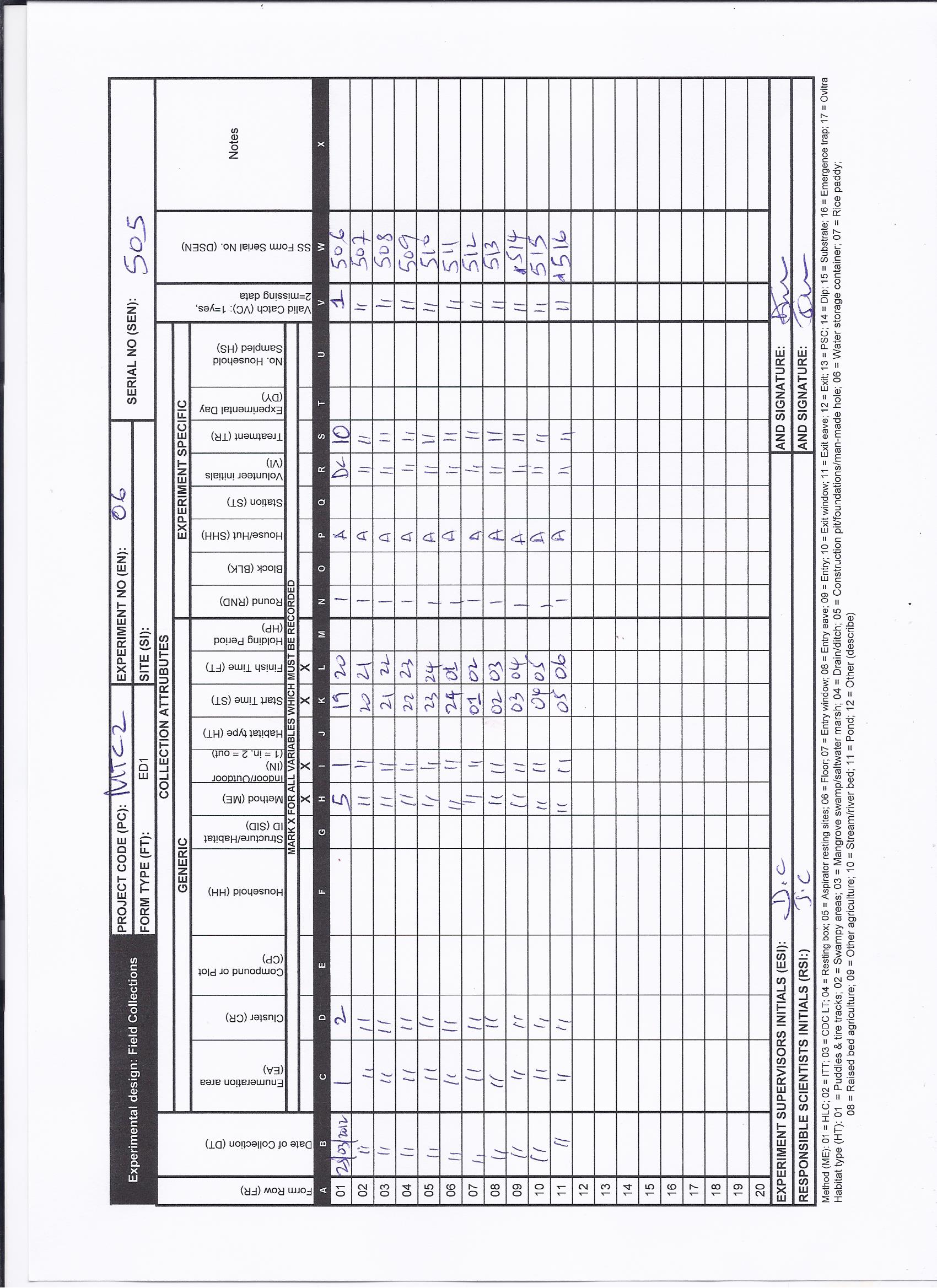
**

The steps involved in the experiment were as follows:

1. **Collect mosquitoes**: One collection is the same as one trapping effort and mosquitoes are placed into one collection cup. Recorded using one line on the experimental design form (ED1) for each hour. Data was recorded for the following collection attributes: d*ate of collection, enumeration area, method, indoor/outdoor, start time, finish time, round, house/hut, volunteer initials, and treatment*. The *treatment*  variable is used to distinguish between one bait to another specifically for this experiment (10 = human, 11 = cow, and 12 = goat). As an example, an ED1 is first filled-in with hourly data information for human as a bait for the first round (Figure S2 24).

Figure S2 25: The SS1 form recording sample sorting corresponding to the first row of ED1, just the first hour, other SS1 forms will be required to record the remaining hours. In this example, a user was able to add taxon that wasn’t pre-printed in the form.

**
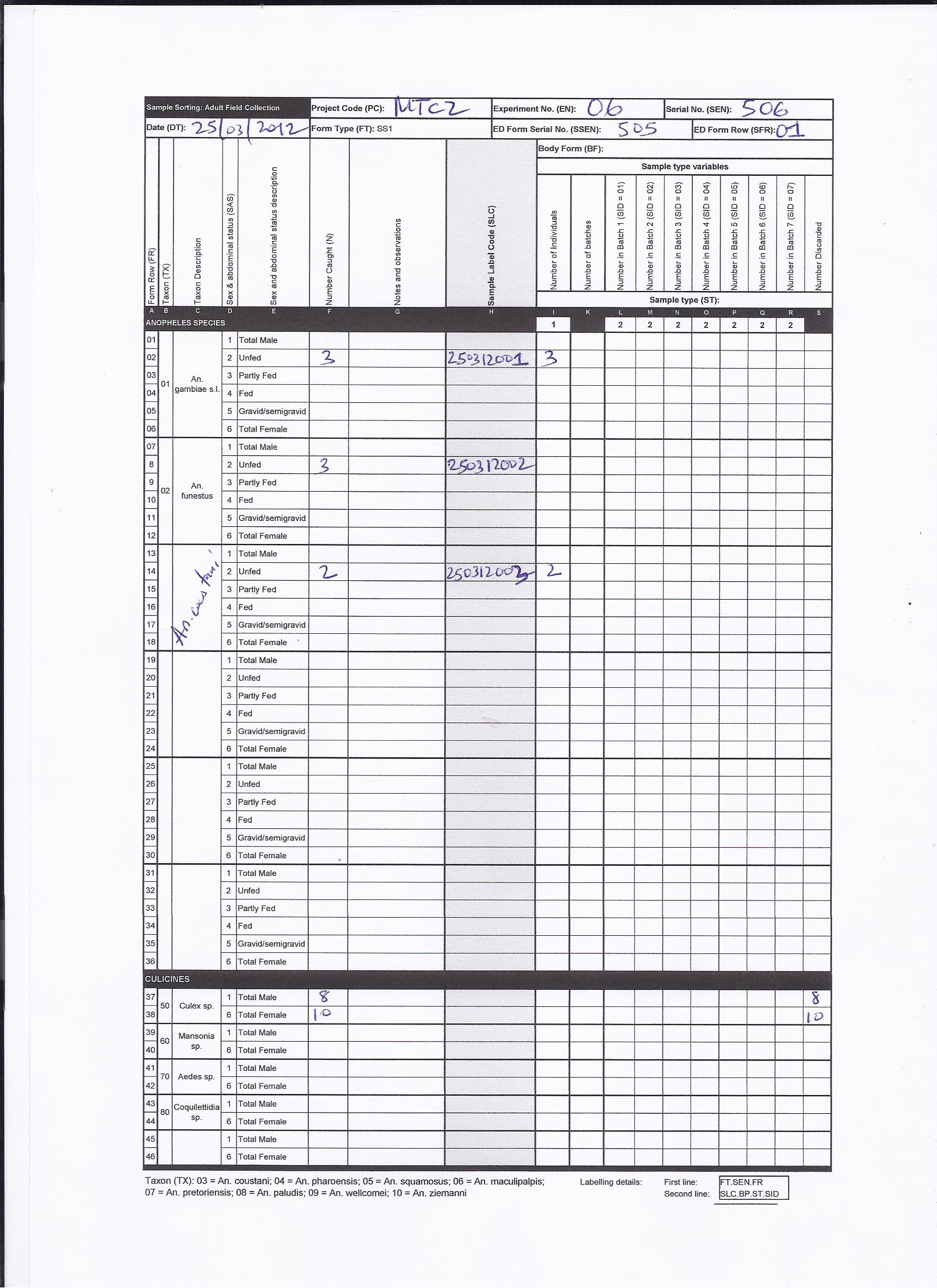
**

1. **Sort collection:** Each collection was sorted into pre-defined subgroups of *taxon, sex* and *abdominal status*. Both these sample sorting attributes are categorical, as follows:
   1. Taxon: *A. gambiae s.l., A. funestus,* Culex spp., Aedes spp., Mansonia spp.
   2. Sex and abdominal status: *total male, unfed, part fed, fed gravid/semigravid, total female.*

The number in each subgroup is counted, and the data from each collection was recorded using one sample sorting form (SS1) Figure S2 25.

In this experiment, culex were discarded, but the sort category, constituent number of specimens, and identity attributes of samples retained for experiments, observations and storage was readily recorded using SO1 for sample observation, and storage form (ST1) just as described for adult mosquitoes in previous examples.
